# A Systems-Wide Analysis of Proteolytic and Lipolytic Pathways Uncovers The Flavor-Forming Potential of The Gram-Positive Bacterium *Macrococcus caseolyticus* subsp. *caseolyticus*

**DOI:** 10.3389/fmicb.2020.01533

**Published:** 2020-07-07

**Authors:** Shahneela Mazhar, Kieran N. Kilcawley, Colin Hill, Olivia McAuliffe

**Affiliations:** ^1^Department of Food Biosciences, Teagasc Food Research Centre, Moorepark, Fermoy, Ireland; ^2^School of Microbiology, University College Cork, Cork, Ireland; ^3^APC Microbiome Institute, Cork, Ireland; ^4^VistaMilk SFI Research Centre, Fermoy, Ireland

**Keywords:** *Macrococcus caseolyticus* subsp. *caseolyticus*, fermented foods, flavor development, whole genome sequence, enzymatic assays, metabolomics

## Abstract

*Macrococcus caseolyticus* subsp. *caseolyticus* is a Gram-positive, commensal organism documented to be present as a component of the secondary microflora in fermented foods such as Ragusano and Fontina cheeses and Cantonese sausage. In these products, the organism appears to play a role in ripening and the development of the final organoleptic qualities. However, the role of this organism in flavor generation is not well understood. Therefore, the objective of this study was to investigate the role of *M. caseolyticus* subsp. *caseolyticus* in flavor compound formation through an examination of enzymatic, metabolomic and genomic data. A bank of *M. caseolyticus* subsp. *caseolyticus* strains derived from a variety of niches were examined. Enzyme activities analyzed comprised those of the proteolytic and lipolytic cascades including cell-envelope proteinase (CEP), peptidases, esterases, lipases, aminotransferases and glutamate dehydrogenase (GDH). Strain to strain variation was observed, often associated with niche. All strains, except those isolated from non-dairy sources, demonstrated high CEP activity. Such high CEP activity associated with dairy strains implies the importance of this characteristic in the adaptation of these strains to a dairy-specific niche. However, limited downstream peptidolytic activity, in addition to a limited ability to generate free amino acids (FAA) was observed across all strains, indicating weak ability of this organism to generate amino-acid derived flavor compounds. Interestingly, the strains with high CEP activity also demonstrated high esterase activity and gas chromatography-mass spectrometry (GC-MS) analysis of the volatile compounds produced when these strains were grown in lactose-free milk demonstrated differences in the range and types of volatiles produced. In contrast to this metabolic versatility, comparative genome analysis revealed the distribution of components of the proteolytic and lipolytic system in these strains to be conserved. Overall, this study demonstrates the potential of *M. caseolyticus* subsp. *caseolyticus* to generate diverse volatile flavor compounds. Additionally, the identification of the highly active strain-specific cell wall bound caseolytic proteases deriving extensive casein hydrolysis, serves as a promising avenue which can be potentially harnessed in the future to produce greater and more diverse flavor compounds.

## Introduction

Members of the *Macrococcus* genus are disseminated in nature as animal commensals and are probable ancestors of staphylococcal species (Hiramatsu et al., 2014). The genus is currently composed of 11 species: *M. bovicus*, *M. carouselicus*, *M. equipercicus*, *M. brunensis*, *M. hajekii*, *M. lamae*, *M. canis*, *M. epidermidis*, *M. goetzii*, *M. bohemicus*, and *M. caseolyticus*, which is further divided into two subspecies: *M. caseolyticus* subsp. *hominis* and *M. caseolyticus* subsp. *caseolyticus* (Mašlaňová et al., 2018). In the early 1900s, strains of *Macrococcus caseolyticus*, at the time known as *Micrococcus caseolyticus*, were isolated from raw milk samples. Early screening of these strains indicated their ability to rapidly and completely peptonize the milk, therefore the name *caseolyticus* (casein-dissolving) was suggested (Evans, 1916). Historically, studies have associated the occurrence of the *M. caseolyticus* species in food systems, as part of the secondary flora of cheese and its role in ripening and flavor development has been documented (Bhowmik and Marth, 1988, 1990). The proteolytic activity of this organism on β-casein was investigated, and shown to lead to the production of short peptides. This suggested that *M. caseolyticus* was capable of producing the substrates for flavor compound production (Moreno and Kosikowski, 1973). The technological applications of enzymes extracted from this organism and their use in the production of novel cheese products have been described in a number of studies and patents (Hargrove and Mcdonough, 1964; Desmazeaud and Hermier, 1968; Alkalaf et al., 1987). The more conventional role of *M. caseolyticus* in the flavor development of Cantonese sausage has also been investigated and the majority of the volatiles generated were identified as metabolites of free fatty acid catabolism (Wu et al., 2009).

The development of flavor in dairy products is a particularly complex process which involves three main processes: glycolysis (sugar metabolism), proteolysis (degradation of proteins) and lipolysis (degradation of lipids) (McSweeney, 2017). Whilst, the metabolisms of sugars mainly lactose, are a source of many flavor compounds, the pathways that are explored in this study are proteolysis and lipolysis. Proteolysis has been regarded as one of the most important processes in the development of flavor and the enzymatic reactions involved are well defined in the lactic acid bacteria (LAB), the group of organisms most widely associated with flavor formation in dairy products (Smit et al., 2005). The proteolytic cascade commences with the breakdown of casein into small peptides by the action of surface bound proteinases, often referred to as cell enveloped proteinases (CEP). The peptides are then transported into the cell and further degraded by the coordinated action of peptidases with different, but often partially overlapping, specificities for amino acids (Gobbetti et al., 2007). The free amino acids (FAA) generated can directly contribute to flavor, but their further metabolism is identified as a key process in flavor formation (McSweeney and Sousa, 2000). There are several pathways that lead to flavor compound generation originating from the catabolism of FAA, initiated by the activity of various enzymes such as aminotransferases (AT), lyases, decarboxylases, deminases, and dehydratases (Jensen and Ardö, 2010). However, the majority of the most important flavor compounds have been reported to originate in the transamination pathway (Jensen and Ardö, 2010). Aminotransferases catalyze the transfer of an amino group to α-keto acid, which are important precursors for the generation of various volatile flavor compounds (Jensen and Ardö, 2010). The transamination reactions are dependent on the presence of an amino group receptor, usually α-ketoglutarate, which is produced by glutamate dehydrogenase (GDH). This key enzyme is described as a rate limiting factor for transamination and therefore essential for the production of precursors which lead to the generation of flavor compounds by members of the LAB (Tanous et al., 2002; Kieronczyk et al., 2004). The lipolytic pathway is also an important process in flavor development and involves the hydrolysis of lipids present in milk to free fatty acids (FFA) and glycerols, mono-or diglycerides by the action of esterases or lipases. The liberation of FFA or short- and intermediate chain fatty acids contribute directly to flavor or serve as precursors for the biosynthesis of numerous flavor-contributing volatile compounds (McSweeney and Sousa, 2000).

The purpose of this study was to investigate the metabolic pathways involved in flavor compound formation in *M. caseolyticus* subsp. *caseolyticus*. Anecdotal evidence suggested that certain strains of this subspecies had a positive impact on the flavor profile of some cheese types. To investigate this further, we analyzed a bank of six *M. caseolyticus* subsp. *caseolyticus* strains (isolated from sources including whale skin, bovine milk and semi-hard cheese) through enzymatic assays, whole genome sequencing and comparative genome analysis, and metabolomics data generated by gas chromatography-mass spectrometry (GC-MS). Additionally, we have investigated FAA and nitrogen utilization capabilities of these strains to further evaluate their capability in producing amino-acid derived flavor compounds. To understand their overall flavor-forming potential, genomic and phenotypic information was coupled with metabolomics data generated by GC-MS and other methodologies.

## Materials and Methods

### Bacterial Strains and Culture Conditions

A total of six *M. caseolyticus* subsp. *caseolyticus* strains were employed in this study and are presented in Table 1. All six strains were cultivated at 37°C for 24 h in Tryptic Soy Broth (TSB; Becton, Dickinson and Company, Berkshire, England). In addition, other control strains were employed for comparative analysis such as *Lactococcus lactis* subsp. *cremoris* Wg2 was used as a positive control for CEP activity and was cultivated in LM17 (Merck, Darmstadt, Germany). *Lactobacillus paracasei* DPC4206 was used as a positive control for general aminopeptidases (PepN, PepC), proline specific dipeptidase (PepX) aromatic aminotransferase (ArAT) and glutamate dehydrogenase (GDH) activities. In addition, *L. paracasei* DPC4536 was also used as a positive control for GDH activity and were cultivated in MRS media (Oxoid, Basingstoke, United Kingdom). *Yarrowia lipolytica* DPC6266 was used as a positive control for lipase activity and was propagated in broth medium containing yeast extract (YE) (Merck, Germany), all control strains were incubated aerobically at 30°C.

**TABLE 1:** Details of the six *M. caseolyticus* subsp. *caseolyticus* strains analyzed in this study.

**Bacterial strain**	**Country**	**Isolated from**	**Isolation Year**	**Accession number**	**References**
ATCC13548^T^	United States	Raw milk	1916	PZJF00000000	Evans, 1916
ATCC51835	NC, United States	Whale skin	1995	SDQL00000000	Kloos et al., 1998
ATCC13518	Unknown	Unknown	1980	ND	Roberts, 1985
DPC6291	Cork, Ireland	Cheese	2017	SDQM00000000	Mazhar et al., 2018, 2019
DPC7170	Cork, Ireland	Cow’s milk	2017	SDQK00000000	Mazhar et al., 2018, 2019
DPC7171	Cork, Ireland	Cow’s milk	2017	SDQJ00000000	Mazhar et al., 2018, 2019

*ND, Not determined.*

### Comparative Genome Analysis and Orthologous Groups Identification

The whole genome sequences (WGS) of five out of the six, *M. caseolyticus* subsp. *caseolyticus* strains are available in the public database and the accession numbers are presented in Table 1. Details of genome sequencing and assembly were previously reported (Mašlaňová et al., 2018; Mazhar et al., 2019). Functional genome distribution (FGD) was conducted on the five *M. caseolyticus* subsp. *caseolyticus* representative strains to identify conserved and non-conserved open reading frame (ORFs) encoding enzymes involved in the proteolytic and lipolytic system using CompACTor v 0.18 at an e-value threshold of 1e^–10^ with FGDfinder v0.022 tool (Altermann, 2012). Additionally, core-genome and singleton analysis was carried out with OrthoVenn (a web platform for orthologous gene clustering) (Wang et al., 2015). Inferred proteins for each of the genomes by Prokka version 1.11 were used as input (Seemann, 2014).

### Qualitative Analysis of Proteolytic and Lipolytic Activity

To evaluate the proteolytic activity of *M. caseolyticus* subsp. *caseolyticus* strains, reconstituted skim milk (RSM) agar was prepared from skim milk powder (Kerry ingredients, Cheshire, United Kingdom) at 10% (w/v) and agar (Agar; Sigma-Aldrich, Wicklow, Ireland) at 1.5% (w/v). The inoculated plates were then incubated for 24 h at 37°C. To determine lipolytic activity, tributyrin agar (Sigma-Aldrich) was prepared according to the manufacturer’s instructions, with the modification prescribed by Bertuzzi (2017). *Y. lipolytica* DPC6266 was used as a positive control. The plates were incubated for 48 h at 37°C. A positive result for protease and lipase activity was scored on the basis of the presence of halo of clearing around the growth of the organism. The test assay was performed in triplicate.

### Quantitative Analysis of the Enzymes Involved in Proteolysis

#### Determination of CEP Activity

Cell envelope proteinase activity was determined using a modification of the method previously described by Stefanovic et al. (2017), which is based on the EnzCheck^®^ kit Green Fluorescence E-6638 (Molecular Probes, Eugene, OR, United States). *M. caseolyticus* subsp. *caseolyticus* strains were grown in 50 ml of TSB for 24 h at 37°C. *L. lactis* subsp. *cremoris* Wg2 used as a positive control and was propagated in 50 ml of LM17 for 24 h at 37°C. Cells were centrifuged (4000 × *g*, 10 min, 4°C), and washed three times with 50 mmol l^–1^ Tris–HCl buffer pH 7.8 with 2 mmol l^–1^ CaCl_2_ added. Components of the kit were prepared according to manufacturer’s instructions; 100 μl of cell suspension and 100 μl of prepared BODIPY^®^ FL casein solution were mixed in 96-well microplate (Sarstedt, Wexford, Ireland) and incubated for 24 h at 37°C. Fluorescence (Ex/Em 505/513 nm) was measured on a Synergy 2 reader (Bio-Tek Multi Detection Plate Reader, Winooski, VT, United States), using optimal filters: 485/20 nm for extinction and 528/20 nm for emission. A proteinase K solution (2 μg ml^–1^) was used as a positive control. Enzyme activities for each strain were expressed as direct fluorescence readings. All strains were evaluated in triplicate. A set of trypsin standards from 0.2 ng ml^–1^ to 70 μg ml^–1^ were prepared and their activity was measured in a similar fashion.

#### Preparation of Cell Free Extract

To obtain cell free extract (CFE), *M. caseolyticus* subsp. *caseolyticus* strains were incubated for 24 h in 50 ml TSB were centrifuged (4000 × *g*, 10 min, 4°C) and washed twice with 50 mmol l^–1^ sodium phosphate buffer pH 7.5 and suspended in the same buffer to a final volume of 5 ml. Cells were disrupted by sonication (Soniprep 150; MSE LTD, London, United Kingdom) in five cycles of 15 s sonication on maximum amplitude (20 amplitude microns) and 45 s of cooling on ice. Sonicated samples were centrifuged (12,000 × *g*, 10 min, 4°C) to remove cell debris. Cell counts (CFU per ml) were evaluated for each strain before and after sonication.

#### Determination of Aminopeptidases, Aminotransferasesm, and Glutamate Dehydrogenase Activities

Aminopeptidases activity was measured according to the method defined by Jensen and Ardö (2010), with the modifications defined by Stefanovic et al. (2017). Chromogenic substrates (L-Lysine p-nitroanilide (pNA) (Sigma-Aldrich), H-Gly-Pro-pNA, H-Arg-pNA, H-Glu-pNA, and H-Ala-Phe-Pro-pNA (Bachem, Bubendorf, Switzerland) for PepN, PepX, PepC, PepA, and PepV, respectively, were prepared as 1 mmol l^–1^ solutions in 50 mmol l^–1^ sodium phosphate buffer pH 7.5. The assay mixture contained 50 μl of substrate solution and 50 μl of CFE. Absorbance was measured at 405 nm (Synergy HT; Bio-Tek Multi Detection Plate Reader) after 60 min of incubation at 37°C. The amount of p-nitroaniline released was determined by including a standard curve obtained for standard samples of p-nitroaniline ranging between 0 and 80 nmol. Aminopeptidase activities were expressed as nmol of p-nitroanilne released per min and mg of protein. *L. paracasei* DPC4206 was used as a control, as its PepN, PepX, and PepC activity was reported by Stefanovic et al. (2017). Blanks contained 50 mmol l^–1^ sodium phosphate buffer instead of CFE. Development of yellow color in the samples, originating from p-nitroaniline, and no color development in the blank after incubation were considered as a sign of enzyme activity of CFE. Protein content was determined by using Qubit ^TM^Protein Assay Kit (Thermo Fisher Scientific, Dublin, Ireland).

Aromatic aminotransferase (ArAT) activity was performed by following the conversion of phenylalanine to phenylpyruvate, as described by Stefanovic et al. (2017). *L. paracasei* DPC4206 was used as a control as its ArAT activity has been reported previously (Stefanovic et al., 2017). Glutamate dehydrogenase (GDH) assay was performed based on the principle described by Kieronczyk et al. (2004), with the modifications defined by Stefanovic et al. (2017). *L. paracasei* DPC4206 and DPC4536 were used as a control as its GDH activity has been described previously (Stefanovic et al., 2017). Protein content was determined as described above and the results were expressed as the number of units of activity per mg of protein. The total quantity of the enzyme that resulted in an increase of absorbance of 0.01 per min corresponded to one unit (U) of activity.

#### Milk Protein Hydrolysis and Free Amino Acid Analysis

To prepare samples for the determination of milk protein hydrolysis and FAA levels, after propagation of *M. caseolyticus* subsp. *caseolyticus* strains for 24 h at 37°C in TSB, cultures were inoculated at 1% in to 20 mls of commercial ultra−high−temperature (UHT) lactose free milk (LFM) (Friendly Farms lactose free milk, Aldi, Ireland). The inoculated LFM (triplicate for each strain) was incubated for 24 h at 37°C. Cell counts were evaluated at time 0 h and 24 h. After 24 h incubation, samples were stored at −20°C and were defrosted at room temperature before the analysis of milk protein hydrolysis and FAA using Reversed−Phase High−Performance Liquid Chromatography (RP−HPLC) and Ion Exchange column, respectively.

RP−HPLC was used to qualitatively assess the extent of hydrolysis of the major milk proteins by the action of cell wall bound proteinases. The analysis was performed according to the method described previously (Mounsey and O’kennedy, 2009). Separation was performed on an Agilent Poroshell 300SB-C18 (75 × 2.1 mm i.d.) column (Agilent Technologies, CA, United States). The HPLC system consisted of an Agilent 1200 Separation Module with DAD Detector and Agilent Chemstation Software. All samples were evaluated in triplicate.

Samples were first subjected to deproteinization for the analysis of FAA as described previously (McDermott et al., 2016), on the soluble N extracts using a Jeol JLC-500/V amino acid analyser (Jeol, Garden city, Herts, United Kingdom) fitted with a Jeol Na^+^ high performance cation exchange column. The chromatographic analysis was conducted at pH 2.2. All samples were evaluated in triplicate and results are expressed as μg ml^–1^ of LFM.

#### Nitrogen Substrate Utilization With Biolog Phenotypic Microarray PM3 Plate

The ability of *M. caseolyticus* subsp. *caseolyticus* strains to utilize a range of nitrogen sources was analyzed with high throughput phenotypic microarrays PM3 plate (Biolog, Hayward, CA, United States) according to the published procedures (Bochner et al., 2001). All reagents and materials for the phenotypic studies were purchased from Biolog (Biolog, United States). The cells from passage four were scrapped from the surface of TSA plates and suspended in PM3 inoculation fluid containing Dye Mix H; 100 μl of a 1:200 dilution of cell suspension at 81% transmittance was added to each well of the PM3 plate. IF-0a GN/GP base inoculating fluid was prepared and plates were inoculated and incubated in the OmniLog incubator for 72 h. Data were collected every 15 min and analyzed using the Biolog Kinetic and Parametric software (Biolog, Hayward, CA, United States). Phenotype diversities were evaluated based on the area differences under the kinetic curves of color formation. The experiment was conducted twice. The data from PM3 was quantitatively analyzed with two modules kinetic and parametric of the Biolog phenotype Microarray software. Area under the curve values (AUC) were extrapolated from the parametric module for each well after subtracting A1 well with kinetic module.

#### Quantitative Analysis of Enzymes Involved in Lipolysis

To determine esterase activity, the conversion of p-nitrophenol butyrate to p-nitrophenol, and butyric acid and p-nitrophenol octanoate to p-nitrophenol, and octanoic acid, was measured using a transparent 96 well microplate (Sarstedt) as described by Bertuzzi (2017). The assay mixture was composed of a buffer (100 mmol l^–1^ sodium phosphate, 150 mmol l^–1^ sodium chloride, 0.5 % v/v triton X-100, at pH 7) and a substrate (50 mmol l^–1^ p-nitrophenol butyrate; 50 mmol l^–1^ p-nitrophenol octanoate in acetonitrile). In each well, 50 μl of buffer, 50 μl of CFE and 10 μl of substrate were mixed and absorbance was measured after 1h of incubation at 37°C at 400 nm (Synergy HT; Bio-Tek Multi Detection Plate Reader). The amount of pnitrophenol released was determined from a standard curve obtained for a set of standards ranging from 0 to 500 nmol of p-nitrophenol. Protein content was determined as described above. The activity was expressed as μmol of p-nitrophenol released per mg of protein.

#### Semi-Quantitative Assay for Hydrolytic Activities (API ZYM)

A range of hydrolytic activities were determined calorimetrically on 19 naphtyl substrates using the API-ZYM kit system (BioMérieux, Hampshire, United Kingdom). The assay was performed according to the manufacturer’s instructions in triplicate.

#### Volatile Compounds Analysis by Gas Chromatography-Mass Spectrometry

LFM inoculated at 1% with *M. caseolyticus* subsp. *caseolyticus* strains as previously described, were used for this analysis and 3 g of each sample was added to a 20 ml amber screw capped La-Pha-Pack headspace vials with silicone/polytetrafluoroethylene septa (Apex Scientific, Kildare, Ireland). Samples were equilibrated to 40°C for 10 min with pulsed agitation of 5 s at 500 rpm using an agitator on a Gerstel MPS autosampler (Anatune, Cambridge, United Kingdom). For this headspace solid phase micro-extraction (HS_SPME) a single 50/30 um Carboxen^TM^/divinylbenzene/polydimethylsiloxane (DVB/CAR/PDMS) fiber was used (Agilent Technologies). The fiber was exposed to the headspace above the samples for 20 min at depth of 1 cm at 40°C with agitation. The fiber was retracted and injected into the GC inlet and desorbed for 2 min at 250°C into a SPL injector with a SPME liner. The fiber was conditioned between runs using a bake out station at 270°C for 3 min using nitrogen to ensure no carry−over between samples. Injections were made on a Shimadzu 2010 Plus GC (Mason Technology Ltd, Dublin, Ireland) with an DB-624 UI (60m x 0.32mm x 1.8μm) column (Agilent Technologies) using a split/splitless injector with a 1:10 split. A merlin microseal was used as the septum (Sigma-Aldrich). The temperature of the column oven was set at 40°C, held for 5 min, increased at 5°C/min to 230°C then increased at 15°C/min to 260°C, held for 5 min yielding at total GC run time of 65 min. Helium was used as a carrier gas held at a constant flow of 1.2 ml/min. The detector was a Shimadzu TQ8030 mass spectrometer detector (Mason Technology, Dublin, Ireland), ran in single quad mode. The ion source temperature was 220°C and the interface temperature was set at 260°C. The MS mode was electronic ionization (70ev) with the mass range m/z scanned between 35 and 250 amu.

All samples were analyzed in the same GC run. A set of external standards (dimethyl sulfide, benzaldehyde, cyclohexanone, butyl acetate, acetone and ethanol (Sigma-Aldrich) at concentrations of 10 ppm were run at the start of the sample set to ensure that both the HS−SPME extraction and MS detection were within specification. Blanks (empty vials) were injected regularly to monitor possible carry−over.

Chromatograms obtained by GC analysis were converted to.cdf format and processed by TargetView^®^ (Markes International, Llantrisant, United Kingdom). Identification of compounds was based on the results of a comparison with the NIST 2011 Mass Spectral Library (Scientific Instrument Services, NJ, United States) and an in−house library produced from external standards (where available) and confirmed by calculating linear retention indices (Van Den Dool and Kratz, 1963).

### Statistical Analysis

All enzymatic assays results were statistically analyzed using one-way analysis of variance (ANOVA) (Biological replicates *n* = 3) with Minitab (Minitab 17, Minitab Inc., Coventry, United Kingdom) followed by least significant difference (LSD) *post hoc test.* ANOVA were also used for testing the significance of differences in PM3, FAA analysis and GC−MS data. Selected substrates from PM3 and total GC−MS volatile profiles were then visualized as heat maps with hierarchical clustering using single linkage in R with the pheatmap package (R Core Team 2015, R Foundation for Statistical Computing, Austria)^1^ and principal component analysis (PCA) was used for the analysis of GC-MS data with R statistical software package (see text footnote 1).

## Results

### High CEP Activity in *M. caseolyticus* subsp. *caseolyticus* Strains Is Associated With a Dairy Origin

**FIGURE 1:**
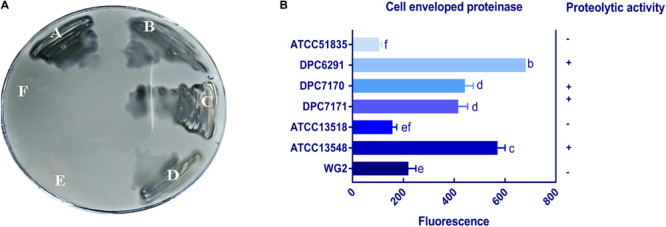
**(A)** Proteolytic action of six *M. caseolyticus* subsp. *caseolyticus* strains on RSM agar medium indicated strains; (A) DPC6291, (B) DPC7170, (C) DPC7171 and (D) ATCC13548 are proteolytic as degradation of the substrate (casein), incorporated in the agar plate by enzyme protease is observed as the development of a transparent zone in these plates in comparison to (E) ATCC13518, and (F) ATCC51835 which were negative after 24 h incubation. **(B)** Cell envelope proteinase (CEP) activities of *M. caseolyticus* subsp. *caseolyticus* as determined by EnzCheck^®^ kit following incubation at 37 °C for 24 h. Bars sharing the same letter show no significant difference according to least significant difference (LSD) test (*p* < 0.05). Strains were analyzed in triplicate. Error bars present standard deviation. The graph presents activities of seven representative strains, including the ATCC 51835 with the lowest activity observed, and *Lactococcus lactis* subsp. *cremoris* Wg2, which was used as a positive control.

Six *M. caseolyticus* subsp. *caseolyticus* strains which were widely distributed both geographically and by the source from which they were isolated (including cheese sample, bovine milk and whale skin) were examined for their proteolytic activities (Table 1). Preliminary screening of the proteolytic activity was determined by examining casein degradation on skim milk agar plates, which is observed as the development of a transparent zone of clearing around the bacterial growth. Of the six strains examined, zones of clearing were observed for strains DPC6291, DPC7170, DPC7171, and ATCC13548, whereas strains ATCC13518 and ATCC51835 were negative for this phenotype after 24 h incubation (Figure 1A). This suggests that while dairy-derived strains were capable of casein degradation on skim milk agar plates, non-dairy associated strains were not.

To quantify the level of CEP activity demonstrated by these strains, a kit based on the proteolysis of BODIPY^®^ FL-labeled casein derivatives was employed. This assay is based on the principle that the measured increase in fluorescence is proportional to the proteinase activity. While all six *M. caseolyticus* subsp. *caseolyticus* strains demonstrated CEP activity when measured by this assay, the levels varied significantly between dairy-derived and non-dairy strains (Figure 1B). The CEP activity was expressed as measured fluorescence and ranged from 130.7 arbitrary fluorescence units for strain ATTC51835, isolated from whale skin, to 682 arbitrary fluorescence units for strain DPC6291, isolated from cheese (Figure 1B), which corresponds to fluorescence measured when standard solutions of trypsin in the range of 0.4–10.0 μg ml^–1^ were used (data not shown). *L. lactis* subsp. *cremoris* Wg2 was used as a CEP-positive control strain, having been confirmed as such in a previous study (Nikolić et al., 2009; Stefanovic et al., 2017). CEP activity for Wg2 was measured at 220 arbitrary fluorescence units. The dairy-derived *M. caseolyticus* strains DPC6291, DPC7170, DPC7171 and ATCC13548 all had statistically higher levels of activity than Wg2 (Figure 1B and Supplementary Table S1).

**FIGURE 2:**
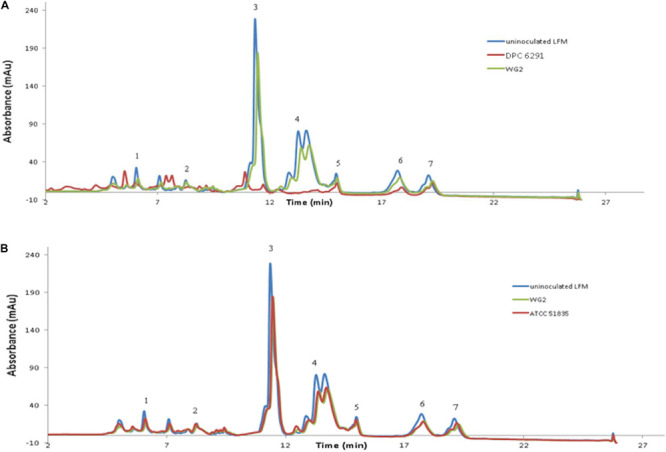
RP-HPLC profiles for the following six strains **(A)** DPC6291, **(B)** ATCC51835 analyzed along with controls uninoculated LFM and *Lactococcus lactis* subsp. *cremoris* Wg2. Peaks representing fraction of casein and whey proteins (1) K-casein, (2) αs2-casein (3) αs1-casein (4) β-casein (5) α-lactalbumin (6) β-lactoglobulin a (7) β-lactoglobulin b.

To confirm the specificity of the observed cell enveloped proteinase activities, reversed-phase high-performance liquid chromatography (RP-HPLC) profiles were generated for each strain following growth in UHT lactose-free milk (LFM). Again, *L. lactis* subsp. *cremoris* Wg2 was used as a CEP-positive control. *M. caseolyticus* subsp. *caseolyticus* DPC6291, isolated from cheese, demonstrated extensive hydrolysis of the majority of the milk and whey proteins (Figure 2A). This was also observed for the other CEP-active strains described above (DPC7170, DPC7171, ATCC13548) (data not shown). In contrast, very little hydrolysis of the milk and whey proteins was observed for strain ATCC51835 (Figure 2B), isolated from whale skin and showing comparatively low CEP activity in the previous assay. This was also the case for ATCC13518, whose source is unknown, and for *L. lactis* subsp. *lactis* WG2. Proteolytic digestion of κ-casein and αS_2_-casein was highest for DPC6291 and varied amongst DPC7170, DPC7171, and ATCC13548, whereas no hydrolysis of these fractions was observed for ATCC51835 and ATCC13518. All strains demonstrated the ability to hydrolyze the αS_1_-casein and β-casein fractions; however, these fractions were predominantly hydrolyzed with the dairy strains DPC6291, DPC7170, DPC 7171, and ATCC13548. Limited activity was observed with ATCC51835 and ATCC13518 against αS_1_-casein and β-casein and peaks for β-casein hydrolysis overlapped and were similar to the control strain Wg2. In fact, ATCC51835 and ATCC13518 strains demonstrated comparatively weak proteolytic activity towards majority of the casein fractions. In addition, the whey proteins preceding the casein fractions were, for the most part, intact across all strains; however, DPC6291 was the only strain to demonstrate moderate ability to hyrdolase β-lactoglobulin. Overall, the extent of hydrolysis of the casein fractions, as observed in this assay, correlated with the activities observed in the CEP assay. The strains showing a high CEP activity, as revealed by the BODIPY^®^ FL-labeled casein assay, illustrated strong affinity towards different fractions of casein, and were derived from dairy-associated sources. In contrast, those with low CEP activity exhibited a low extent of hydrolysis of casein (Figure 2B) and were from non-dairy sources as in the case of ATCC51835, derived from whale skin, or in the case of ATCC13581, the source of which is unknown.

### Limited Downstream Proteolytic Enzyme Activity in *M. caseolyticus* subsps *caseolyticus* Strains in Comparison to LAB Flavor Formers

**FIGURE 3:**
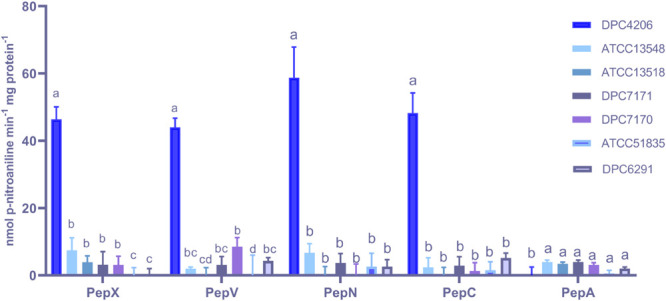
Aminopeptidase (PepX, PepV, PepN, PepC, and PepA) activities of six *M. caseolyticus* subsp. *caseolyticus* (ATCC13548, ATCC13518, DPC7171, DPC7170, DPC6291 and ATCC51835) along with *Lactobacillus paracasei* DPC4206 determined by measuring cleavage of corresponding chromogenic substrates (L-Lys-pNA, Arg-pNA and Gly-Pro-pNA, H-Glu-pNA and H-Ala-Phe-Pro-pNA) for PepN, PepC and PepX, PepA and PepV, respectively. Results are expressed as nmol of released p-nitroaniline min^–1^ mg protein^–1^. Bars sharing the same letter show no significant difference according to least significant difference (LSD) test (*P* < 0.05). Strains were analyzed in triplicate. Error bars present standard deviation.

The activities for PepN, PepX, PepC, PepA, and PepV peptidases were examined in the *M. caseolyticus* subsp. *caseolyticus* strain bank. *L. paracasei* DPC4206 was used as a positive control for activities of PepN, PepX, and PepC as these had been previously reported (Stefanovic et al., 2017). Surprisingly, all *M. caseolyticus* strains showed very limited activities towards the various substrates tested (Figure 3). The PepN activities measured ranged from 0.3 to 6.7 nmol para-nitroaniline (per min per mg protein) for DPC6291 and ATCC13548, respectively. PepX activities ranged from 3.07 for DPC7170 to 7.43 nmol para-nitroaniline (per min per mg protein) for ATCC13548, while, PepC activities ranged from 1.26 for DPC7170 to 5.14 nmol para-nitroaniline (per min per mg protein) for DPC6291. PepV activities ranged from 3.09 for DPC7171 to 8.53 nmol para-nitroaniline (per min per mg protein) for DPC7170 and PepA activities ranged from 0.59 to 5.43 nmol para-nitroaniline (per min per mg protein) for ATCC 51835 and ATCC13548, respectively. PepN, PepC, PepX, and PepV activities expressed by the positive control strain DPC4206 were in the range of 58.7, 48.23, 46.4, and 44.02 nmol para-nitroaniline (per min per mg protein), respectively. In all cases (except PepA), the activities of the peptidases exhibited by the *M. caseolyticus* subsp. *caseolyticus* strains were significantly lower in comparison to the *L. paracasei* DPC4206, a LAB strain with proven peptidolytic ability (Figure 3 and Supplementary Table S1).

To examine the ability of the strains to generate FAA, the levels of FAA in LFM milk following fermentation with each of the six *M. caseolyticus* subsp. *caseolyticus* strains were analyzed with HPLC. The analysis indicated no significant (*P* < 0.05) differences between the test strains and the uninoculated control-LFM, except for the amino acids histidine, proline, and tryptophan (Supplementary Figure S1 and Supplementary Table S1). The most significant difference was the release of tryptophan by the protease active strains, highest in DPC6291 of 103.22 μg ml^–1^/lactose free milk. These results suggest that the *M. caseolyticus* subsp. *caseolyticus* strains tested, irrespective of source, display poor peptidolytic activity and as a result, cannot generate significant levels of FAA when provided with a rich protein source such as milk.

**FIGURE 4:**
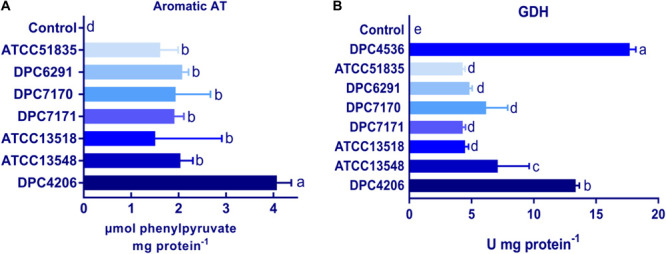
**(A)** Aromatic aminotransferase (ArAT) activities of *M. caseolyticus* subsp. *caseolyticus* strains along with *Lactobacillus paracasei* DPC4206 determined by measuring the absorbance of phenylpyruvate, the final product of transamination between phenylalanine and α-ketoglutarate. Results are expressed as μmol of released phenylpyruvate/ (min*mg of protein). **(B)** Glutamate dehydrogenase (GDH) activities of strains of *M. caseolyticus* subsp. *caseolyticus* along with *Lactobacillus paracasei* DPC4206 and DPC 4536 by following change in absorbance during a reaction catalyzed by GDH enzyme in which glutamic acid is converted to α-ketoglutarate in the presence of NAD+. Results are presented as Units of enzyme activity per mg of protein, where the unit represents the amount of enzyme giving an increase of absorbance of 0.01 per 1 min. Bars sharing the same letter show no significant difference according to least significant difference (LSD) test (*P* < 0.05). Strains were analyzed in triplicate. Error bars present standard deviation.

The activities of the other downstream enzymes with an important role in the proteolytic cascade leading to flavor compound formation were also examined. When ArAT activity was measured, the *M. caseolyticus* subsp. *caseolyticus* strains showed significantly lower levels of activity when compared to *L. paracasei* DPC4206 which was used as positive control strain as its ArAT activity has been previously reported (Stefanovic et al., 2017). The ArAT activity expressed by DPC4206 in this study was 4.07 μmoles of phenylpyruvate released per mg protein (slightly higher than the previous study). The activity of ArAT measured for *M. caseolyticus* subsp. *caseolyticus* ranged from 1.5 μmoles of phenylpyruvate released per mg protein for ATCC13518 to 2.07 μmoles of phenylpyruvate released per mg protein for DPC6291. Statistical analysis revealed no significant differences between the *M. caseolyticus* subsp. *caseolyticus* strains for ArAT activity (Figure 4A). The GDH activity of the strains was also analyzed and compared to *L. paracasei* strains DPC4206 and DPC4536, strains whose GDH activities had been determined in previous study (Stefanovic et al., 2017). The GDH activity for *M. caseolyticus* subsp. *caseolyticus* strains ranged from 4.2 for strains ATCC51835 and ATCC7171 to 7.1 U mg^–1^ of protein for ATCC13548. The GDH activity for the control strains DPC4206 and DPC4536 ranged from 13.3 to 17.3 U mg^–1^ of protein (correlating with previous report) and differences in GDH activities were shown to be significant (Figure 4B). Overall, the ArAT and GDH activities measured across the *M. caseolyticus* subsp. *caseolyticus* strains were significantly lower than the LAB strains tested in this study.

The ability of the *M. caseolyticus* subsp. *caseolyticus* strain bank to metabolize a diverse range of nitrogen sources was examined with high throughput PM3 plates from Biolog (Biolog, CA, United States). Overall, the six strains examined showed little activity, as they were only able to metabolize 13.6% of the nitrogen sources tested, the majority of which were fatty acids (e-amino-N-caproic acid, D,L-a-amino-caprylic acid, D-amino-N-valeric acid) and di-peptides (Ala-His, Gly-Glu, Met-Ala). This further confirms the limited ability of the members of this subspecies to catabolize FAA. Differences in AUC for all substrates were analyzed using ANOVA and activities demonstrated no significant differences between strains. A detailed list of the AUC values of each substrate in each well of PM3 along with ANOVA analysis can be found in Supplementary Table S1. The heat map in Supplementary Figure S2 illustrates the substrates most effectively metabolized by these strains in the PM3 plate.

### High Esterase Activity Observed in Strains With High CEP Activity

**FIGURE 5:**
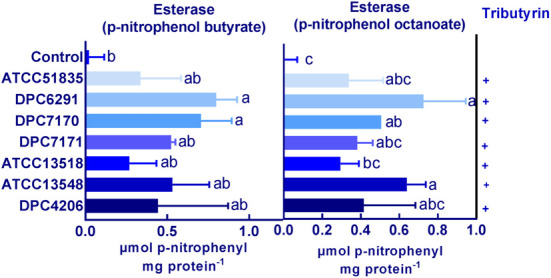
Esterase activity on p-nitrophenol butyrate and p-nitrophenol octanoate for the six *M. caseolyticus* subsp. *caseolyticus* strains along with DPC 4206 are presented. Activity is expressed in μmol of p-nitrophenol released per mg of protein^–1^. Bars sharing the same letter show no significant difference according to least significant difference (LSD) test (*P* < 0.05). Strains were analyzed in triplicate. Error bars present standard deviation. Tributyrin hydrolysis: positive; +.

The breakdown of lipids also plays an important role in flavor development. Lipase activity for the *M. caseolyticus* subsp. *caseolyticus* strains were qualitatively analyzed on tributyrin agar as outlined by Bertuzzi (2017). All strains demonstrated weak hydrolytic activity in comparison to the control strain *Y. lipolytica* DPC6266 on tributyrin agar (data not shown). Meanwhile, esterase activity was measured using both p-nitrophenol butyrate and p-nitrophenol octanoate as substrates. The highest activity towards p-nitrophenol butyrate was obtained with DPC6291 of 0.79 μmol of p-nitrophenol released per mg of protein and lowest with ATCC51835 of 0.33 μmol of p-nitrophenol released per mg of protein. Likewise, the highest activity towards p-nitrophenol octanoate was observed in DPC 6291 of 0.72 μmol of p-nitrophenol released per mg of protein and lowest with ATCC 51835 of 0.33 μmol of p-nitrophenol released per mg of protein (Figure 5). The esterase activities were statistically significant and comparatively higher amongst the high CEP active strains (DPC6291, DPC7170, DPC7171, and ATCC13548). The esterase activity of dairy related *M. caseolyticus* subsp. *caseolyticus* strains was also significant in comparison to the *L. paracasei* DPC4206.

Additionally, hydrolytic activities towards a number of lipase, esterase, proteinase and peptidase substrates were evaluated for all the *M. caseolyticus* subsp. *caseolyticus* strains along with *L. paracasei* DPC4206 and *Y. lipolytica* DPC6266 determined using the semi-quantitative API-ZYM kit system. All *M. caseolyticus* subsp. *caseolyticus* strains were positive for esterase activity (C4:0), and weakly positive for esterase (C8:0), whereas *Y. lipolytica* DPC 6266 was positive towards both and DPC4206 was negative for both esterase (C4:0) and esterase (C8:0). All *M. caseolyticus* subsp. *caseolyticus* strains were negative for lipase, peptidase, trypsin and α-chymotrypsin like proteinase and glycosidase, in comparison to *L. paracasei* DPC4206 which demonstrated hydrolytic activities of the peptidase enzymes leucine arylamidase, valine arylamidase, and cystine arylamidase. Glycosidase and phosphatase activities were also observed in DPC4206. *Y. lipolytica* DPC6266 was also positive for leucine arylamidase, acid phosphatase, phosphohydrolyase and β-glucosidase, and, negative for lipase (C14) using this assay (Supplementary Table S2). The results from this analysis further confirm the lipolytic activity towards butyrate (C4), whereas no peptidolytic activity was observed across the members of the *M. caseolyticus* subsp. *caseolyticus*.

### Comparative Genomics Reveals the Conservation of the Proteolytic and Lipolytic System Components in *M. caseolyticus* subsp. *caseolyticus* Strains

A systematic genome-wide analysis of components of the proteolytic and lipolytic systems from five draft genomes of strains from the *M. caseolyticus* subsp. *caseolyticus* strain bank was conducted. FGD was performed on all of five strains to identify ORFs specific to each of the strains and also ORFs that were conserved between these strains (Altermann, 2012). Mining of conserved genes within the five genomes using FGD at an e-value of 1e^–10^ with zero mismatches identified 1,314 conserved ORFs. The numbers of non-conserved genes varied from 113 to 150 for each of the strains. Subsequently, the presence of ORFs encoding genes for enzymes involved in proteolysis and lipolysis was examined across all five genomes. Interestingly, irrespective of the niche from which they were derived, the distribution of the genes encoding the components of the proteolytic and lipolytic processes in *M. caseolyticus* subsp. *caseolyticus* appeared to be conserved across strains (a detailed list of genes with GI codes can be found in Supplementary Table S3). The number of genes encoding the proteinases, peptide transporters, peptidases, aminotransferases, dehydrogenases, lyases, lipases, and esterases are shown in Table 2. The distribution of proteolytic components such as the cell wall bound proteinase (*prt*P) and the presence of oligopeptide (OPP) transport gene cluster (opp*ABCDF*) were widely distributed across all five genomes. The general broad specificity peptidases, PepN and PepC that are repoted to be widely distributed across LAB were not identified (Liu et al., 2010). Conversely, the presence of exopeptidases with different substrate specificities such as PepA that has a narrow activity towards only acidic amino acids (Glu and Asp substrates), PepP (a proline-specific peptidase), PepM (a methionine-specific peptidase), PepV (broad specificity dipeptidase), PepT (capable of hydrolyzing only tripeptides), carboxypeptidases and endopeptidases such as PepF were identified across the five genomes. Further, genes encoding the downstream enzymes of the proteolytic pathways involved in the transamination processes, which includes ATs (*ilv*E, *roc*D, *his*C, *glm*S) and GDH (*gdh*A) were found to be conserved across the five studied genomes. The distributions of lipolytic enzymes such as monoacylglycerol lipase (*mgl*P and *mgI*L) which are responsible for the hydrolysis of monoacylglycerol into free fatty acid and glycerol, and carboxylesterase which catalyze the hydrolysis of various types of esters were also widely distributed across the five genomes.

**TABLE 2:** Distribution of enzymes responsible for flavor development in selected *Macrococcus caseolyticus* subsp. *caseolyticus* genomes.

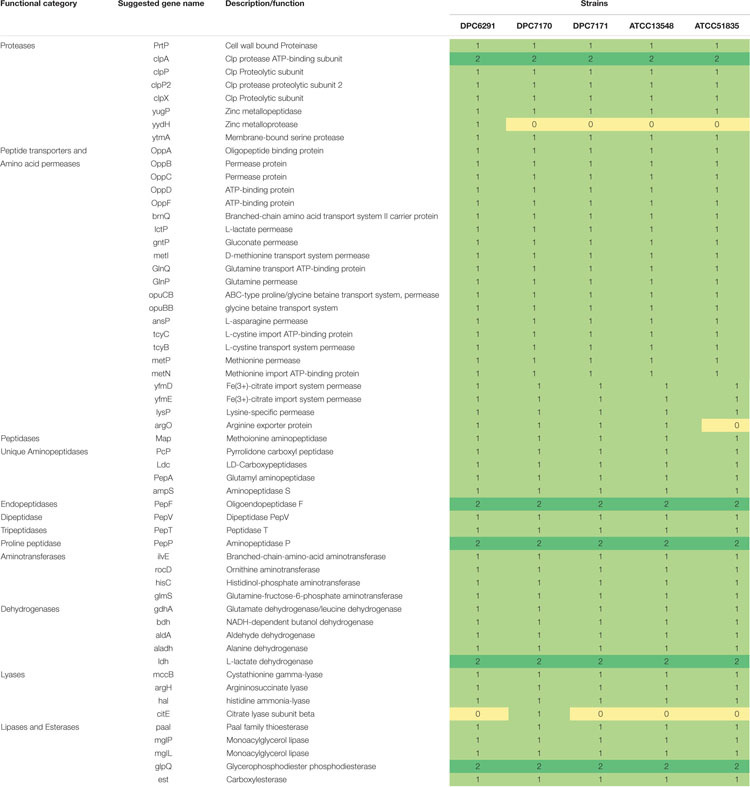

*The number of identified genes is indicated. Color shading shows absence (yellow) and presence single gene (light green) or multiple genes (dark green).*

**FIGURE 6:**
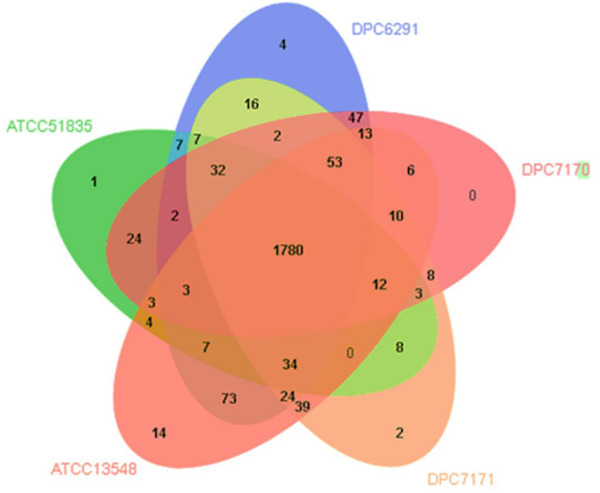
Venn diagram showing shared orthologous protein clusters amongst the five *Macrococcus caseolyticus* subsp. *caseolyticus* strains. A total of 2238 clusters were identified, of which 462 were orthologous and 1776 single copy gene cluster identified with the default parameters, 1e–5 e-value cutoff for all protein similarity comparisons and 1.5 inflation value for the generation of orthologous clusters. The numbers in the diagram indicate overlapped conserved gene clusters or un-overlapped specific gene clusters in every single strain.

Additionally, OrthoVenn 2 was used identify the distribution of shared orthologous clusters and singletons across the five genomes (Wang et al., 2015). According to this web-based software, the genomes shared 2,238 clusters constituting pan-genome, whereas, the core-genome represented in all strains was estimated in 1,780 clusters (Figure 6), whose functions were mostly assigned to metabolic processes including the proteolytic and lipolytic catabolic pathways (a detailed list of core orthologous clusters with Gene ontology ID can be found in Supplementary Table S4). The accessory genome, composed of singleton gene clusters unique to each strain, were composed of genes whose functions were mostly unknown, or associated with virulence factors, resistance determinants and mobile genetic elements (MGEs) such as transposons, phages and plasmid proteins. In summary, both the FGD and OrthoVenn 2 comparative genomic analysis of the five strains did not reveal genetic content differences with regards the components of the proteolytic and lipolytic cascade, except for the presence of gene *yyd*H encoding a zinc metalloprotease belonging to MEORPS family M50b in DPC6291 and citrate lyase subunit beta in DPC7170.

### Metabolomic Analysis With GC-MS Separates *M. caseolyticus* subsp. *caseolyticus* in to Two Distinct Groups Based on High/Low CEP and Esterase Activity

HS-SPME GC-MS was used to analyze the production of volatile flavor compounds generated as a result of the metabolic activities of the selected *M. caseolyticus* subsp. *caseolyticus* strains in LFM milk after 24 h incubation. A total of 74 volatile compounds were detected in the samples including the control, of which the majority were esters (35.1%) followed by ketones (16.2%), alcohols (6.7%), aldehydes (5.4%), benzenes (5%), and some sulfurs (4.1%). Twenty-three of these volatiles were only present in the test samples. Specifically, these were twelve esters, two ketones, one benzene, two aldehydes, one alcohol, one sulfur, two acids, and two phenols (Supplementary Table S5).

**FIGURE 7:**
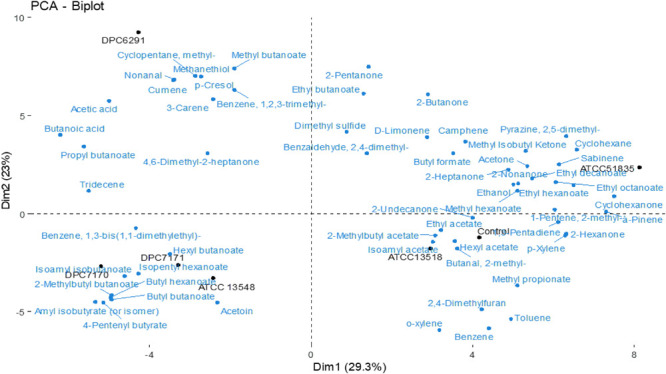
Principle component analysis (PCA) Bioplot illustrates the compounds responsible for the separation between test strains after 24 h incubation in LFM detected via HS-SPME GC-MS. Uninionculated LFM is used as a control.

The PCA bioplot based on the volatiles detected describes 29.3 and 23% total variation between the first and second component, respectively. There is a clear separation of the strains from the control except for strain ATCC13518 (Figure 7). DPC6291 which has a very high CEP and esterase activity was completely separated from the rest of the strains; its position was associated with relative high levels of esters, methyl ketones, straight chain aldehyde, fatty acids and nitrogen, sulfur, and phenol compounds (pyrazines, sulphurs and phenol; derived from FAA catabolism). Some of these compounds at a lower abundance were also present in the control. Overall, DPC6291 was significantly (*P* < 0.05) associated with the production of methyl butanoate, propyl butanoate, nonanal, methanethiol, acetic acid, butanoic acid (derived from lipolysis), and p-cresol (Supplementary Table S5). The relatively high abundance of these fatty acid esters, straight chain aldehydes and nitrogen compounds act as a dominating factor in the discrimination of DPC6291 from other strains.

DPC7170, DPC7171, and ATCC13548 strains were positioned together and were linked to numerous esters and some methyl ketones (Figure 7). The three strains were significantly (*P* < 0.05) associated with the production of hexyl butanoate, isopentyl hexanoate, butyl hexanoate, butyl butanoate, isoamyl isobutanoate, 2-methylbutyl butanoate, 4-pentenyl butyrate, and amyl isobutyrate. The abundance of ester compounds originating from microbial esterification of different acids (derived via lipolysis, oxidation of amino acids or glycolysis) act as a main factor in separating these strains (Supplementary Table S5).

Also, the strains with comparatively low CEP and esterase actitivtiy, ATCC51835 and ATCC13518 were positioned separately from each other (Figure 7). ATCC51835 was separated from the other strains and was associated with abundance (*P* < 0.05) of esters and a ketone such as ethyl decanoate, methyl hexanoate and 2-undecanone (fatty acid oxidation). ATCC13518 was positioned with the control uninionculated LFM and was mainly associated with abundance (*P* < 0.05) of benzeneacetaldehyde (precursor phenylalanine), phenylethyl alcohol (precursor phenylalanine) 3-methyl butanal (FAA metabolism) and 2,3 heptanedione (FFA metabolism) (Supplementary Table S5). Altogether, these volatiles are associated with wide range of classes (esters, benzenes, aldehydes and ketones) which act as a dominating factor and separates these low CEP and esterase active strains ATCC51835 and ATCC13518 from the comparatively highly active strains (DPC6291, DPC7170, DPC7171, and ATCC13548).

**FIGURE 8:**
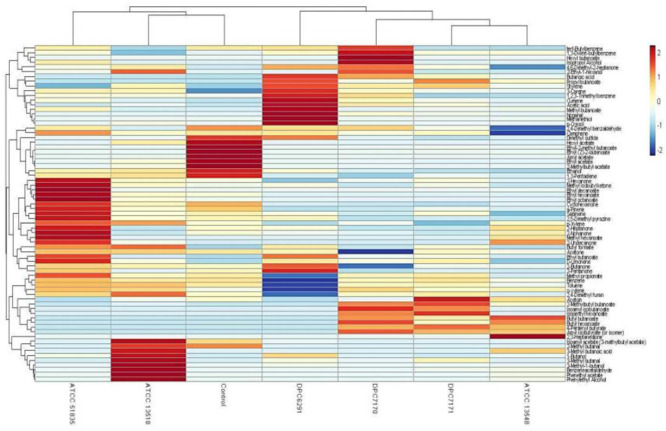
Hierarchical clustered heat map illustrates Trial 1 result of relative abundance of variable compounds separating high CEP and esterase active (ATCC13548, DPC7171, DPC7170, and DPC6291) strains from those with comparatively low CEP and esterase activity (ATCC 135185 and ATCC 51835) *M. caseolyticus* subsp. *caseolyticus* after 24 h incubation in LFM detected via HS-SPME GC-MS.

A hierarchical clustered heat map illustrates the relative abundance of variable compounds associated with different strains correlates with the PCA’s illustrating the clear separation of high CEP and esterase active strains forming a separate clade (ATCC6291, DPC7170, DPC7171, and ATCC13548) from those with relatively low activity (ATCC13518 and ATCC51835) clustering closely with the control (Figure 8).

The cell enumerations of *M. caseolyticus* subsp. *caseolyticus* strains in LFM pre and post-incubation are presented in Supplementary Table S6. Compounds selected as flavor contributing which were absent in the control were according to previously published reviews of compounds considered mainly as flavor contributors in cheeses are presented in Supplementary Table S7 (Curioni and Bosset, 2002).

## Discussion

Flavor is result of a combination of both taste and aroma, and the volatiles responsible for the typical flavor and aroma of fermented products are produced mainly by the metabolism of proteins, fats and carbohydrates. Of these, proteolysis is identified as particularly important for flavor development in fermented dairy products and the components of this pathway have been well defined in LAB (Liu et al., 2010). These dairy-associated microorganisms have been extensively used in food fermentations as flavor generators. However, currently, there is a drive to examine the metabolic diversity of strains that might not normally be associated with dairy products, as such strains may serve as a tools for the production of novel and distinct flavor profiles (McAuliffe et al., 2019). Therefore, in this study, we have explored the metabolic potential of *M. caseolyticus* subsp. *caseolyticus* to contribute to flavor formation. This species is found to be associated with certain fermented food types and has been documented to have a history of safe use and potential technological benefit according to the International Dairy Federation (IDF) Inventory of Microbial Food Cultures (Bourdichon et al., 2012), although information regarding its specific role in flavor generation is limited.

Prior to the commencement of this study, we had isolated a single strain of *M. caseolyticus* subsp. *caseolyticus* (DPC6291) from semi-hard cheese. Preliminary examination of the proteolytic capability of this strain was examined on RSM agar, and indicated rapid and extensive casein degradation after an overnight incubation. Similarly, *M. caseolyticus* subsp. *caseolyticus* ATCC13548 strain had been investigated previously by Bhowmik and Marth in 1988, which demonstrated complete and rapid degradation of β-casein and other fractions of casein, suggested the ability of this strain to generate flavor and aroma-forming compounds (Bhowmik and Marth, 1988). In an effort to examine the flavor forming potential of this subspecies, we established a strain bank of *M. caseolyticus* subsp. *caseolyticus* from a variety of sources as described previously (Mazhar et al., 2018), and performed a systems-wide analysis of the pathways potentially contributing to flavor formation in fermented foods.

The cell-enveloped proteinases, encoded by *prt*P and its homologs, plays an important role in flavor development, as its activity towards casein hydrolysis results in the provision of substrates for the subsequent steps of the proteolytic cascade. Our genome-wide comparative analysis on the five *M. caseolyticus* subsp. *caseolyticus* out of the six strains from our strain bank revealed the presence of a single copy of *prt*P across all of the five strains examined (DPC6291, DPC7170, DPC7171, ATCC13548, and ATCC51835). However, the CEP activity of the six *M. caseolyticus* subsp. *caseolyticus* strains analyzed in this study indicated strain-to strain variability with dairy-derived strains displaying extensive capability in hydrolyzing casein (DPC6291, DPC7170, DPC7171 and ATCC13548). This variability was also evident when RP-HPLC profiles were examined, which clearly demonstrated the extensive hydrolysis of casein fractions with high CEP-active, dairy-associated strains (DPC6291, ATCC13548, DPC7170, and DPC7171) and comparatively lower activity towards casein fractions were observed with a less CEP-active, non-dairy derived strain ATCC51835, and also with strain ATCC13518, the source of which is unknown. In addition, the high protease action of the dairy-derived strains was apparent on RSM plates and when these strains were inoculated in LFM milk leading to coagulation of the milk after an overnight incubation at pH values (between the range 5.15 – 5.72) above those defined for acid-induced coagulation (pH ∼4.6) (Phadungath, 2005). This coagulation was absent in less CEP-active strains, which further highlights the extensive milk protein hydrolysis capabilities of the high CEP-active strains. Overall, the presence of *prt*P homologs across the dairy and the non-dairy associated genomes did not correlate with the phenotype. Further examination of the amino acid sequence encoded by the *prt*P gene indicated comparatively high sequence similarities amongst the dairy derived strains (>99%) and relatively less sequence similarities between the dairy and the non-dairy derived strain ATCC51835 (∼98%). These mutations in the *prt*P gene could be the cause of the variability obtained in the phenotypic expression of the proteinase. The genome analysis has also identified the presence of CLP ATPases proteases across the five genomes which are reported to be active towards caseins (Gottesman et al., 1990; Bertuzzi, 2017). Therefore, the significant protease activity observed in dairy derived strains could be either as a result of the action of caseolytic protease CEP (*prt*P) or a combined action with the CLP ATPases proteases.

The presence of peptide transporters oligo-peptide (OPP) across all five genomes suggests the ability for peptide uptake and their subsequent metabolism in these strains. However, other transport systems which are well described in LAB such as Di/tripeptide (DtpT/DtpP) were not identified, implying a limitation in the uptake of nitrogen sources in the form of dipeptides or tripeptides and the subsequent utilization of such peptides by these organisms (Liu et al., 2010). The next step after oligo-peptide uptake in proteolysis is the internal hydrolysis of peptides to FAA by the action of peptidases. In this study, all six *M. caseolyticus* subsp. *caseolyticus* strains demonstrated limited activity towards general broad specificity and proline specific peptidases which correlates with the absence of genes encoding for these enzymes in the genome. All six strains were further analyzed for their activity towards Glu and Ala-Phe-Pro-pNA substrates for PepA and PepV peptidases, which were identified in the genomes. However, all strains demonstrated limited activity towards these substrates indicating either these enzymes may not be efficient or the substrates used are not appropriate to evaluate their activity.

A previous investigation conducted by our group with a bank of *Lactobacillus casei* strains, established that ArAT was a suitable test case for the determination of general AT activity (Stefanovic et al., 2017). Therefore, in this study, we investigated the ArAT activities across the six strains as a representation of the general AT activity. The presence of number of AT genes in the genome correlated with the phenotypic data, which revealed that all tested strains demonstrated ArAT activity. However, the activity was comparatively lower than *L. paracasei* DPC4206 (positive control strain). GDH activity was detected in all strains with ATCC13548 as statistically significant; however the level of activity was lower in comparison to *L. paracasei* DPC4206 and DPC4536 and other strains of LAB reported in previous studies (Stefanovic et al., 2017), but corresponds to the activity reported for *Staphylococcus saprophyticus* strain DPC5671 and *M. caseolyticus* DPC6291 investigated by Bertuzzi (2017).

Moreover, FAA and Phenotypic microarray PM3 analysis conducted further confirmed limited peptidyl hydrolases capability of the six strains. The evaluation of FAA release in fermentates of the six test strains indicated a significant release of only histidine, proline and tryptophan out of 20 amino acids analyzed, in comparison to the control. From the PM3 analysis, it was revealed that strains were capable of metabolizing only 13.6% of the total nitrogen sources tested, of which the majority were fatty acids (e-Amino-N-Caproic acid, D,L-a-Amino-Caprylic acid, D-Amino-N-Valeric acid) and di-peptides (Ala-His, Met-Ala, Gly-Glu). The hydrolysis di-peptides which may correlate with the presence of the PepV and PepA peptidases present in the genomes. The broad specificity dipeptidase activity of PepV may act on the Ala-His and Met-Ala substrates while the glutamyl aminopeptidase PepA may act on the Gly-Glu substrate (Christensen et al., 1999). In addition, in all six strains, L-cysteine was the most significantly metabolized nitrogen source, correlating with the presence of the *suf*S gene, encoding cysteine desulfurase, an enzyme that transforms L-cysteine to L-alanine and S-sulfanylcysteine, in all five genomes. Therefore, L-cysteine could represent one of the few preferred nitrogen sources required for the growth of this organism (Supplementary Table S1).

Altogether, our conclusion from our enzyme and genomic analysis is that the proteolytic system of *M. caseolyticus* subsp. *caseolyticus* differs considerably from those of the well described LAB species. Their ability to use the peptide substrates generated from casein is constrained by their limited peptidolytic activity. The absence of general peptidases and the weak activities of those present along with lower dehydrogenase (GDH) and aminotransferase (ArAT) activities required for the catabolism of amino acids suggests an inability of this organism to catabolize a wide array of amino acids and therefore, to produce significant quantities of amino acid-derived flavor compounds. The implications of this in a mixed strain culture system, such as in the manufacture of hard and semi-hard cheese, is that these enzymes are most likely provided by other strains in the mix, thus complementing the limited proteolytic activity of *M. caseolyticus* subsp. *caseolyticus*.

Comparative-genome analysis also indicated the presence of genes encoding lipase enzymes such as monoacylglycerol lipase (*mgl*P and *mgI*L) and carboxylesterase. The phenotypic data correlated with the genomic data as the lipase and esterolytic activity was observed in all strains. However, the esterase activity was significant in strain DPC6291 and DPC7170, and was higher than the *L. paracasei* DPC4206 strain. The esterase activity of DPC6291 on p-nitrophenol butyrate has been previously reported to be the most significant amongst the highly lipolytic strain *Y. lipolytica* DPC6266 by Bertuzzi (2017), and our results correlate with this study. Another study also identified the volatile compounds in cantonese sausage inoculated with a strain of *M. caseolyticus* subsp. *caseolyticus* to originate mainly from degradation and oxidation of lipids (Wu et al., 2009). The enzymatic analysis, together with genomic analysis and the utilization of FFA in the PM3 nitrogen plates (capracylic, valeric, and caproic acid), indicate the capability of this organism to metabolize FFA.

Finally, the volatile profiles of the six strains inoculated in LFM milk presented some associations with the enzymatic activities analyzed. A majority of the compounds absent in the uninionculated LFM milk control were straight and branched chain esters, compound’s considered to be metabolites of FFAs. Specifically, DPC6291, ATCC13548, DPC7170, and DPC7171 demonstrated significant esterase activity, whereas, all strains demonstrated lipolytic activity on tributyrin agar. Interestingly, the high CEP active strain DPC6291 was associated with the production of the phenol compound, p-cresol and the production of the sulfur compound methanethiol. The generation of p-cresol may be associated with the ability of DPC6291 to metabolize tryptophan as identified in FAA analysis, whereas the production of methanethiol could be associated with enzymes present in the genome such as PepM (methionine-specific activity) which functions to remove N-terminal methionine residues from proteins and cystathionine γ-lyase which catalyzes the production of methanethiol from methionine or they could have been produced from the precursors or contaminants present in the milk. These compound have been listed as main odorant in number of cheeses such as British farmhouse cheddar, smear ripened and mold ripened cheeses (Molimard and Spinnler, 1996; McSweeney and Sousa, 2000; Curioni and Bosset, 2002). DPC6291 was also associated with the production of the aldehyde nonanal (derived from β-oxidation of FFAs), which is associated with green, citrus and fatty aroma identified in soft cheeses (Sablé and Cottenceau, 1999; Collins et al., 2003). ATCC13548, DPC7170, and DPC7171 were significantly associated with the production of esters. The key odorants produced by these strains included hexyl butanoate, isopentyl hexanoate, butyl hexanoate, butyl butanoate which are linked with sweet, fruity, and floral notes (Curioni and Bosset, 2002). The majority of the compounds identified in the relatively low protease and esterase active strains, ATCC 51835 and ATCC 13518 were also present in the control but in significantly lower amounts. Therefore, in the hierarchical clustering, these strains cluster more closely to the control, ATCC13518 being the most similar. There was one compound identified as a potent odorant absent in control and significantly (*P* < 0.05) produced only by ATCC13518, 3-methyl-butanal (leucine transamination) associated with green, malty aroma. This compound could have originated as a result of methylenation of butanal present in the control. 3-methyl-butanal is identified as a potent odorant in Camembert aged cheddar and in a number of other cheese varieties (Griffith and Hammond, 1989; Curioni and Bosset, 2002). In comparison to other strains, a limited number of volatiles were significantly associated with ATCC51835 (*n* = 3). One of these is a ketone, 2-undecanone, identified as key aroma compounds in Camembert cheese, and reported to originate from fatty acid oxidation (Curioni and Bosset, 2002; Bertuzzi et al., 2018). Overall, the volatile analyses of *M. caseolyticus* subsp. *caseolyticus* strains confirms their metabolic diversity as they demonstrated different capacities for the production of flavor compounds correlating with the enzymatic analysis as majority of these compounds are mainly associated with FFA metabolism. Hierarchical clustering of the high CEP and esterase active strain demonstrates that these dairy derived strains are more metabolically active in LFM milk, sharing several similar volatile compounds as these strains cluster together and sperate from the control (uninionculated LFM milk), whereas the non-dairy derived strains with relatively low CEP and esterase activity sharing similar profile with the control and were also comparatively less metabolically active in LFM milk, therefore separated and clustered together.

## Conclusion

This study demonstrates the genomic, phenotypic ability and the metabolic diversity of six *M. caseolyticus* subsp. *caseolyticus* strains for the production of flavor compounds. The genomic comparison revealed the components of the proteolytic and lipolytic system to be conserved. On the other hand, the observed variability in their activities especially in case of CEP and esterases activities indicates that these may be a consequence of different regulation and not due to the different number of key enzyme encoding homologs. The high CEP activity of *M. caseolyticus* subsp. *caseolyticus* strains resulting in extensive casein hydrolysis indicates the ability of these strains to generate high levels of substrates to feed the subsequent steps in the proteolytic cascade. However, limited peptidase activity was observed correlating with the absence of general peptidases in the genomes of these strains. Overall, *M. caseolyticus* subsp. *caseolyticus* demonstrated the ability to generate diverse volatiles with some potent odorants, which makes them potentially useful for further investigation as adjuncts. Work is ongoing to examine the potential of the *M. caseolyticus* subsp. *caseolyticus* strains in combination with high peptidolytic LAB strains, this synergistic effect on the metabolism of proteins may potentially enhance generation of flavor compounds originating from the proteolytic pathway. However, a note of caution should be advised as our genome analysis has also revealed the presence of multiple antibiotic resistance and virulence genes in members of this subspecies (unpublished data). While these specific genes may be considered niche adaptation factors, they could potentially contribute to these organisms being unsuitable for food applications and this information should be considered on a case-by-case in any future use in food systems.

## Data Availability Statement

All datasets presented in this study are included in the article/Supplementary Material.

## Author Contributions

SM and OM designed the work. SM carried out the genomic phenotypic and enzymatic analysis of the strains and wrote the manuscript. KK conducted the volatile analysis. CH reviewed the manuscript. All authors contributed to the article and approved the submitted version.

## Conflict of Interest

The authors declare that the research was conducted in the absence of any commercial or financial relationships that could be construed as a potential conflict of interest.

## Footnotes

1 https://R-project.org

## Supplementary Material

FIGURE S1 Free amino acid analysis of the six *M. caseolyticus* subsp. *caseolyticus.* All samples were evaluated in triplicate. Results are expressed as μg ml^–1^ of lactose free milk. Bars sharing the same letter show no significant difference according to least significant difference (LSD) test (*p* < 0.05).

FIGURE S2 Heat map of substrates that were most effectively metabolized by *M. caseolyticus* subsp. *caseolyticus* strains in PM3 plate.

TABLE S1 Results of the least significant test (LSD) (*p* < 0.05) performed after Analysis of Variance (ANOVA) for all enzymatic analyses of strains of *M. caseolyticus* subsp. *caseolyticus* in enzymatic assays [cell envelope proteinase (CEP), aminopeptidases PepN, PepC, PepX, PepA and di-peptidase PepV, aromatic aminotransferase (ArAT), glutamate dehydrogenase (GDH)], Free amino acid analysis and PM3 analysis.

TABLE S2 Semi-Quantitative assay of enzyme activities of *M. caseolyticus* subsp. *caseolyticus* and control strains.

TABLE S3 Enzyme genes responsible for flavor generation in *Macrococcus caseolyticus* (GI codes are indicated). Color shading shows no gene (white), a single gene (yellow), or multiple genes (green).

TABLE S4 A detailed list of core orthologous clusters with Gene ontology ID for the five strains.

TABLE S5 Volatiles detected with HS-SPME GCMS absent in control and associated with test strains, reported with relative identification CAS number and the linear retention index (LRI). All results presented are obtained in this study.

TABLE S6 Cell enumeration presented in log CFU ml^–1^, in Lactose free milk before (*t* = 0 h) and after incubation at 37°C (*t* = 24 h). pH values; in LFM after incubation at 37°C (*t* = 24 h).

TABLE S7 Flavor contributing Volatiles significantly associated with *M. caseolyticus* subsp. *caseolyticus* strains (*P* < 0.05).
