# Supplementary figures and images for: A Systems-Wide Analysis of Proteolytic and Lipolytic Pathways Uncovers The Flavor-Forming Potential of The Gram-Positive Bacterium Macrococcus caseolyticus subsp. caseolyticus
Source: Front Microbiol. 2020 Jul 7;11:1533. doi: 10.3389/fmicb.2020.01533 (PMC7358451; doi:10.3389/fmicb.2020.01533)

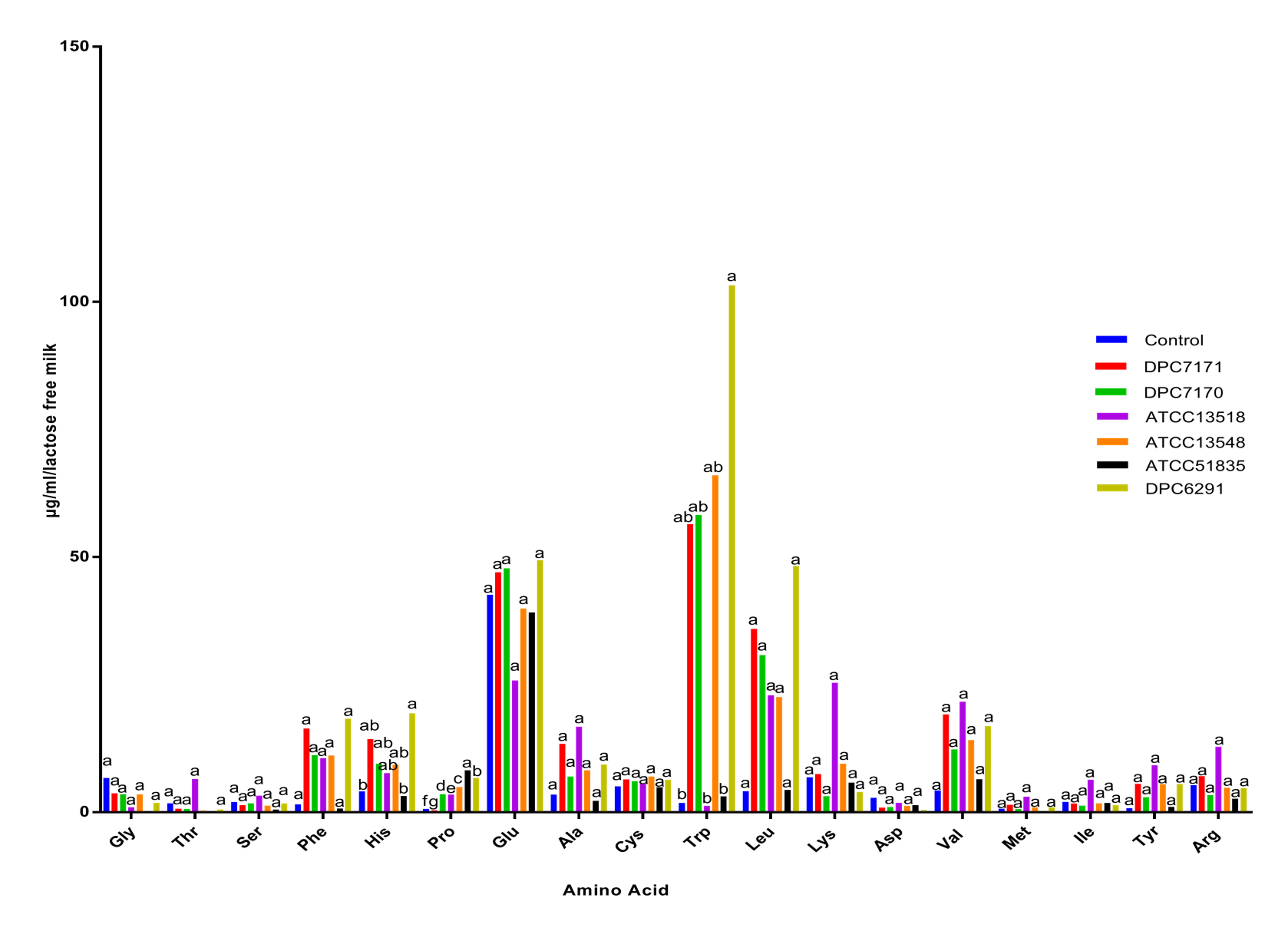

Supplement: FIGURE S1 — Free amino acid analysis of the six M. caseolyticus subsp. caseolyticus. All samples were evaluated in triplicate. Results are expressed as μg ml–1 of lactose free milk. Bars sharing the same letter show no significant difference according to least significant difference (LSD) test (p < 0.05). [file Image_1.TIF]

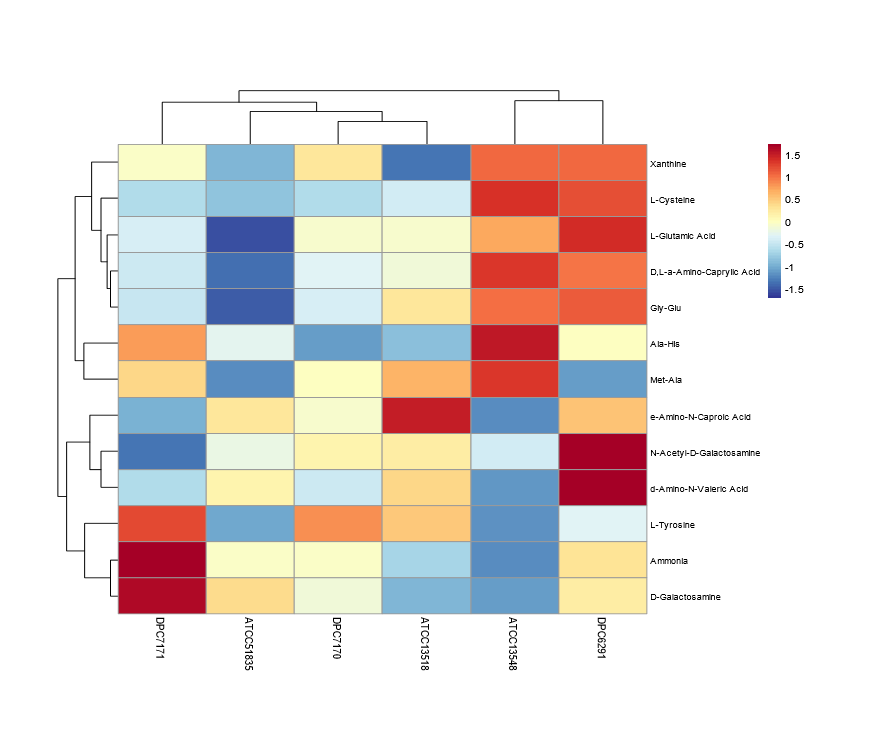

Supplement: FIGURE S2 — Heat map of substrates that were most effectively metabolized by M. caseolyticus subsp. caseolyticus strains in PM3 plate. [file Image_2.TIF]
