# Supplementary material for: A Systems-Wide Analysis of Proteolytic and Lipolytic Pathways Uncovers The Flavor-Forming Potential of The Gram-Positive Bacterium Macrococcus caseolyticus subsp. caseolyticus
Source: Front Microbiol. 2020 Jul 7;11:1533. doi: 10.3389/fmicb.2020.01533 (PMC7358451; doi:10.3389/fmicb.2020.01533)
Supplement: TABLE S1 — Results of the least significant test (LSD) (p < 0.05) performed after Analysis of Variance (ANOVA) for all enzymatic analyses of strains of M. caseolyticus subsp. caseolyticus in enzymatic assays [cell envelope proteinase (CEP), aminopeptidases PepN, PepC, PepX, PepA and di-peptidase PepV, aromatic aminotransferase (ArAT), glutamate dehydrogenase (GDH)], Free amino acid analysis and PM3 analysis. [file Table_1.DOCX]

Supporting Information Table S1: Results of the least significant test (LSD) (p<0.05) performed after Analysis of Variance (ANOVA) for all enzymatic analyses of strains of *M. caseolyticus* subsp *caseolyticus* in enzymatic assays (cell envelope proteinase (CEP), aminopeptidases PepN, PepC, PepX, PepA and di-peptidase PepV, aromatic aminotransferase (ArAT), glutamate dehydrogenase (GDH)), Free amino acid analysis and PM3 analysis.

CEP (Cell enveloped activities)

| DPC/ATCC strains and controls | Fluorescence | Stat. different groups |
| --- | --- | --- |
| Positive control Prot K (1 μg/mL) | 1141.1 | A |
| 6291 | 682.11 | B |
| 13548 | 570.4 | C |
| 7170 | 442.5 | D |
| 7171 | 416.2 | D |
| Wg2 | 220.36 | E |
| 13518 | 157.69 | EF |
| 51835 | 105.99 | F |

PepX (Aminopeptidase X) activities:

| DPC strain or sample | nmol p-NA/(min*mg protein) | Stat. different groups |
| --- | --- | --- |
| 6291 | -29.53 | C |
| 13548 | 7.43 | B |
| 7170 | 3.07 | B |
| 7171 | 3.14 | B |
| 4206 | 46.4 | A |
| 13518 | 3.9 | B |
| 51835 | -30.13 | C |

PepN (Aminopeptidase N) activities:

| DPC strain or sample | nmol p-NA/(min*mg protein) | Stat. different groups |
| --- | --- | --- |
| 6291 | 0.32 | B |
| 13548 | 6.71 | B |
| 7170 | -1.96 | B |
| 7171 | 3.7 | B |
| 4206 | 58.75 | A |
| 13518 | -22.4 | B |
| 51835 | 2.59 | B |

PepC (Aminopeptidase C) activities:

| DPC strain or sample | nmol p-NA/(min*mg protein) | Stat. different groups |
| --- | --- | --- |
| 6291 | 5.147 | B |
| 13548 | 2.357 | B |
| 7170 | 1.26 | B |
| 7171 | 2.867 | B |
| 4206 | 48.23 | A |
| 13518 | -0.15 | B |
| 51835 | 1.54 | B |

PepV (Dipeptidase pepV) activities

| DPC strain or sample | nmol p-NA/(min*mg protein) | Stat. different groups |
| --- | --- | --- |
| 6291 | 4.296 | BC |
| 13548 | 1.965 | BC |
| 7170 | 8.53 | B |
| 7171 | 3.09 | BC |
| 4206 | 44.02 | A |
| 13518 | -11.31 | CD |
| 51835 | -18.93 | D |

PepA (Aminopeptidase A) activities:

| DPC strain or sample | nmol p-NA/(min*mg protein) | Stat. different groups |
| --- | --- | --- |
| 6291 | 2 | A |
| 13548 | 5.43 | A |
| 7170 | 3.08 | A |
| 7171 | 3.96 | A |
| 4206 | -21.66 | B |
| 13518 | 3.441 | A |
| 51835 | 0.59 | A |

ArAT (aromatic aminotransferase) activites:

| DPC strain or sample | µmol Ph-pyruvate/mg protein | Stat. different groups |
| --- | --- | --- |
| 6291 | 2.07 | B |
| 13548 | 2.04 | B |
| 7170 | 1.93 | B |
| 7171 | 1.91 | B |
| 4206 | 4.073 | A |
| 13518 | 1.50 | B |
| 51835 | 1.61 | B |

GDH (glutamate dehydrogenase) activities

| DPC strain or sample | U/mg protein | Stat. different groups |
| --- | --- | --- |
| 6291 | 4.8373 | D |
| 13548 | 7.17 | C |
| 7170 | 6.17 | D |
| 7171 | 4.299 | D |
| 4206 | 13.3604 | B |
| 13518 | 4.484 | D |
| 51835 | 4.29 | D |
| 4536 | 17.3936 | A |

Esterase (p-nitrophenol octanoate) activities

| DPC strain or sample | p-nitrophenol/mg protein | Stat. different groups |
| --- | --- | --- |
| 6291 | 0.726 | A |
| 13548 | 0.6397 | AB |
| 7170 | 0.507889 | AB |
| 7171 | 0.3816 | ABC |
| 4206 | 0.416 | A B C |
| 13518 | 0.2945 | BC |
| 51835 | 0.338 | ABC |

Esterase (p-nitrophenol butyrate) activities

| DPC strain or sample | p-nitrophenol/mg protein | Stat. different groups |
| --- | --- | --- |
| 6291 | 0.7988 | A |
| 13548 | 0.532 | AB |
| 7170 | 0.705 | A |
| 7171 | 0.5231 | AB |
| 4206 | 0.443 | AB |
| 13518 | 0.2686 | AB |
| 51835 | 0.337 | AB |

Free amino acid analysis (Ion Exchange column)

| Amino acid: | **Cysteic acid** |  |
| --- | --- | --- |
| DPC and ATCC strains | µg/ml | Stat. different groups |
| Control (Lactose free milk) | 9.55475 | A |
| DPC7171 | 14.33875 | A |
| DPC7170 | 17.53675 | A |
| ATCC13518 | 36.97 | A |
| ATCC13548 | 17.405 | A |
| ATCC51835 | 12.3875 | A |
| DPC6295 | 22.456 | A |
| Amino acid: | **Taurine** |  |
| DPC and ATCC strains | µg/ml | Stat. different groups |
| Control (Lactose free milk) | 14.357 | A |
| DPC7171 | 8.9795 | A |
| DPC7170 | 16.122 | A |
| ATCC13518 | 15.3305 | A |
| ATCC13548 | 16.21725 | A |
| ATCC51835 | 15.5885 | A |
| DPC6295 | 11.49325 | A |
| Amino acid: | **Aspartic acid** |  |
| DPC and ATCC strains | µg/ml | Stat. different groups |
| Control (Lactose free milk) | 2.82225 | A |
| DPC7171 | 0.89275 | A |
| DPC7170 | 1.01175 | A |
| ATCC13518 | 1.82125 | A |
| ATCC13548 | 1.2115 | A |
| ATCC51835 | 1.411 | A |
| DPC6295 | 0.434 | A |
| Amino acid: | **Glycine** |  |
| DPC and ATCC strains | µg/ml | Stat. different groups |
| Control (Lactose free milk) | 6.68675 | A |
| DPC7171 | 3.69 | A |
| DPC7170 | 3.557 | A |
| ATCC13518 | 0.96125 | A |
| ATCC13548 | 3.5015 | A |
| ATCC51835 | 0.0115 | A |
| DPC6295 | 1.82525 | A |
| Amino acid: | **Threonine** |  |
| DPC and ATCC strains | µg/ml | Stat. different groups |
| Control (Lactose free milk) | 1.7745 | A |
| DPC7171 | 0.68675 | A |
| DPC7170 | 0.6625 | A |
| ATCC13518 | 6.492 | A |
| ATCC13548 | 0.289 | A |
| ATCC51835 | 0.196 | A |
| DPC6295 | 0.5425 | A |
| Amino acid: | **Serine** |  |
| DPC and ATCC strains | µg/ml | Stat. different groups |
| Control (Lactose free milk) | 1.7745 | A |
| DPC7171 | 0.68675 | A |
| DPC7170 | 0.6625 | A |
| ATCC13518 | 6.492 | A |
| ATCC13548 | 0.289 | A |
| ATCC51835 | 0.196 | A |
| DPC6295 | 0.5425 | A |
| Amino acid: | **Phenylalanine** |  |
| DPC and ATCC strains | µg/ml | Stat. different groups |
| Control (Lactose free milk) | 1.53775 | A |
| DPC7171 | 16.40175 | A |
| DPC7170 | 11.15575 | A |
| ATCC13518 | 10.5765 | A |
| ATCC13548 | 11.13875 | A |
| ATCC51835 | 0.71375 | A |
| DPC6295 | 18.3515 | A |
| Amino acid: | **Histidine** |  |
| DPC and ATCC strains | µg/ml | Stat. different groups |
| Control (Lactose free milk) | 4.108 | B |
| DPC7171 | 14.3575 | AB |
| DPC7170 | 9.46825 | AB |
| ATCC13518 | 7.65425 | AB |
| ATCC13548 | 9.318 | AB |
| ATCC51835 | 3.16575 | B |
| DPC6295 | 19.3655 | A |
|  |  |  |
| Amino acid: | **Proline** |  |
| DPC and ATCC strains | µg/ml | Stat. different groups |
| Control (Lactose free milk) | 0.657 | F |
| DPC7171 | 0.388 | G |
| DPC7170 | 3.536 | D |
| ATCC13518 | 3.444 | E |
| ATCC13548 | 4.9415 | C |
| ATCC51835 | 8.215 | A |
| DPC6295 | 6.7195 | B |
|  |  |  |
| Amino acid: | **Glutamic acid** |  |
| DPC and ATCC strains | µg/ml | Stat. different groups |
| Control (Lactose free milk) | 42.6195 | A |
| DPC7171 | 46.999 | A |
| DPC7170 | 47.81625 | A |
| ATCC13518 | 25.83 | A |
| ATCC13548 | 39.9245 | A |
| ATCC51835 | 39.14375 | A |
| DPC6295 | 49.39325 | A |
|  |  |  |
| Amino acid: | **Alanine** |  |
| DPC and ATCC strains | µg/ml | Stat. different groups |
| Control (Lactose free milk) | 3.4705 | A |
| DPC7171 | 13.39625 | A |
| DPC7170 | 6.97925 | A |
| ATCC13518 | 16.74775 | A |
| ATCC13548 | 8.22175 | A |
| ATCC51835 | 2.231 | A |
| DPC6295 | 9.349 | A |
| Amino acid: | **Cysteine** |  |
| DPC and ATCC strains | µg/ml | Stat. different groups |
| Control (Lactose free milk) | 5.07725 | A |
| DPC7171 | 6.4275 | A |
| DPC7170 | 6.07325 | A |
| ATCC13518 | 5.53175 | A |
| ATCC13548 | 6.98675 | A |
| ATCC51835 | 4.89825 | A |
| DPC6295 | 6.3425 | A |
|  |  |  |
| Amino acid: | **Tryptophan** |  |
| DPC and ATCC strains | µg/ml | Stat. different groups |
| Control (Lactose free milk) | 1.80675 | B |
| DPC7171 | 56.475 | AB |
| DPC7170 | 58.232 | AB |
| ATCC13518 | 1.22825 | B |
| ATCC13548 | 66.0055 | AB |
| ATCC51835 | 3.08775 | B |
| DPC6295 | 103.2255 | A |
|  |  |  |
| Amino acid: | **Leucine** |  |
| DPC and ATCC strains | µg/ml | Stat. different groups |
| Control (Lactose free milk) | 4.1415 | A |
| DPC7171 | 35.95575 | A |
| DPC7170 | 30.77825 | A |
| ATCC13518 | 22.93525 | A |
| ATCC13548 | 22.57125 | A |
| ATCC51835 | 4.36325 | A |
| DPC6295 | 48.256 | A |
|  |  |  |
| Amino acid: | **Lysine** |  |
| DPC and ATCC strains | µg/ml | Stat. different groups |
| Control (Lactose free milk) | 6.848 | A |
| DPC7171 | 7.494 | A |
| DPC7170 | 3.152 | A |
| ATCC13518 | 25.38 | A |
| ATCC13548 | 9.523 | A |
| ATCC51835 | 5.804 | A |
| DPC6295 | 3.985 | A |
|  |  |  |
| Amino acid: | GABA |  |
| DPC and ATCC strains | µg/ml | Stat. different groups |
| Control (Lactose free milk) | 0 | A |
| DPC7171 | 0.163 | A |
| DPC7170 | 0.05325 | A |
| ATCC13518 | 66.40025 | A |
| ATCC13548 | 0.03075 | A |
| ATCC51835 | 0.03175 | A |
| DPC6295 | 0.15175 | A |
|  |  |  |
| Amino acid: | **Valine** |  |
| DPC and ATCC strains | µg/ml | Stat. different groups |
| Control (Lactose free milk) | 4.2715 | A |
| DPC7171 | 19.17275 | A |
| DPC7170 | 12.31175 | A |
| ATCC13518 | 21.6525 | A |
| ATCC13548 | 14.1495 | A |
| ATCC51835 | 6.48675 | A |
| DPC6295 | 16.9245 | A |
|  |  |  |
| Amino acid: | **Methionine** |  |
| DPC and ATCC strains | µg/ml | Stat. different groups |
| Control (Lactose free milk) | 0.7115 | A |
| DPC7171 | 1.464 | A |
| DPC7170 | 0.62325 | A |
| ATCC13518 | 3.02525 | A |
| ATCC13548 | 0.924 | A |
| ATCC51835 | 0.1665 | A |
| DPC6295 | 0.93625 | A |
|  | AAcid |  |
| Amino acid: | **Isoleucine** |  |
| DPC and ATCC strains | µg/ml | Stat. different groups |
| Control (Lactose free milk) | 2.01775 | A |
| DPC7171 | 1.72175 | A |
| DPC7170 | 1.28225 | A |
| ATCC13518 | 6.3645 | A |
| ATCC13548 | 1.77775 | A |
| ATCC51835 | 1.79475 | A |
| DPC6295 | 1.42 | A |
|  |  |  |
| Amino acid: | **Tyrosine** |  |
| DPC and ATCC strains | µg/ml | Stat. different groups |
| Control (Lactose free milk) | 0.80575 | A |
| DPC7171 | 5.55375 | A |
| DPC7170 | 2.96625 | A |
| ATCC13518 | 9.21125 | A |
| ATCC13548 | 5.4825 | A |
| ATCC51835 | 1.04375 | A |
| DPC6295 | 5.50575 | A |
|  |  |  |
| Amino acid: | **Arginine** |  |
| DPC and ATCC strains | µg/ml | Stat. different groups |
| Control (Lactose free milk) | 5.331 | A |
| DPC7171 | 7.077 | A |
| DPC7170 | 3.352 | A |
| ATCC13518 | 12.85 | A |
| ATCC13548 | 4.8 | A |
| ATCC51835 | 2.668 | A |
| DPC6295 | 4.739 | A |

Differences in nitrogen substrate utilisation using Area under the curve values tested with Analysis of Variance (ANOVA) for each well of the PM3 plate:

| Well: | **A02** |  | **A03** |  | **A04** |  | **A05** |  |
| --- | --- | --- | --- | --- | --- | --- | --- | --- |
| **Substrates:** | **Ammonia** | | **Nitrite** | | **Nitrate** | | **Urea** | |
| DPC and ATCC strains | AUC values | Stat. different groups | AUC values | Stat. different groups | AUC values | Stat. different groups | AUC values | Stat. different groups |
| 7170 | 2979 | A | 1638.5 | A | -975 | A | -420.5 | A |
| 7171 | 5930 | A | 2438.5 | A | -1172.5 | A | -1090.5 | A |
| 13518 | 1860 | A | 445 | A | -354.5 | A | -82 | A |
| 13548 | 1002 | A | -186.5 | A | -2180 | A | -1811.5 | A |
| 51835 | 2937.5 | A | 2591 | A | -1028.5 | A | 640 | A |
| 6291 | 3549.5 | A | 1955 | A | 270 | A | 336.5 | A |
|  | | | | | | | | |
| **Well:** | **A06** |  | **A07** |  | **A08** |  | **A09** |  |
| Substrates: | **Biuret** | | **L-Alanine** | | **L-Arginine** | | **L-Asparagine** | |
| DPC and ATCC strains |  | Stat. different groups | AUC values | Stat. different groups | AUC values | Stat. different groups | AUC values | Stat. different groups |
| 7170 | -982.5 | A | -4308 | A | -1638.5 | A | -2428 | A |
| 7171 | -4337.5 | A | -725 | A | -1709.5 | A | 607 | A |
| 13518 | -313.5 | A | -5869 | A | 954 | A | -25 | A |
| 13548 | -1828 | A | -1276 | A | -835.5 | A | 1688.5 | A |
| 51835 | -1557 | A | -5086.5 | A | 2453 | A | 739 | A |
| 6291 | -810.5 | A | 3248 | A | 1494.5 | A | 3207 | A |
|  | | | | | | | | |
| **Well:** | **A10** |  | **A11** |  | **A12** |  | **B01** |  |
| Substrates: | **L-Aspartic Acid** | | **L-Cysteine** | | **L-Glutamic Acid** | | **L-Glutamine** | |
| DPC and ATCC strains | AUC values | Stat. different groups | AUC values | Stat. different groups | AUC values | Stat. different groups | AUC values | Stat. different groups |
| 7170 | -542.5 | A | 6850 | A | 2578 | A | -1309 | A |
| 7171 | -2454 | A | 6804.5 | A | 1877.5 | A | -1088.5 | A |
| 13518 | 3435.5 | A | 7739.5 | A | 2601 | A | -857.5 | A |
| 13548 | 2007.5 | A | 14768 | A | 4379.5 | A | -404.5 | A |
| 51835 | 1901.5 | A | 5995 | A | -719 | A | 745 | A |
| 6291 | 5126.5 | A | 14146 | A | 5866.5 | A | 5216 | A |
| Well: | **B02** |  | **B03** |  | **B04** |  | **B05** |  |
| Substrates: | **Glycine** | | **L-Histidine** | | **L-Isoleucine** | | **L-Leucine** | |
| DPC and ATCC strains | AUC values | Stat. different groups | AUC values | Stat. different groups | AUC values | Stat. different groups | AUC values | Stat. different groups |
| 7170 | -6597 | A | -6740 | A | -4289 | A | 398.5 | A |
| 7171 | -595.5 | A | -943 | A | -2921.5 | A | 2047.5 | A |
| 13518 | -3975.5 | A | -8919.5 | A | -6256.5 | A | -2367 | A |
| 13548 | 1732.5 | A | -5237 | A | -2917.5 | A | -1021.5 | A |
| 51835 | -4466 | A | -1845 | A | -9259 | A | -4147 | A |
| 6291 | -2227 | A | -3717.5 | A | -2559.5 | A | -2458.5 | A |
|  | | | | | | | | |
| Well: | **B06** |  | **B07** |  | **B08** |  | **B09** |  |
| Substrates: | **L-Lysine** | | **L-Methionine** | | **L-Phenylalanine** | | **L-Proline** | |
| DPC and ATCC strains | AUC values | Stat. different groups | AUC values | Stat. different groups | AUC values | Stat. different groups | AUC values | Stat. different groups |
| 7170 | -398.5 | A | 1065 | A | -3180 | A | -84.5 | A |
| 7171 | 718 | A | 4438.5 | A | 1692.5 | A | 1828 | A |
| 13518 | -2199.5 | A | -612.5 | A | -2717 | A | 2951 | A |
| 13548 | -4410.5 | A | -1123 | A | -3896 | A | -491 | A |
| 51835 | 2235.5 | A | -1059 | A | -742.5 | A | 1547.5 | A |
| 6291 | 183 | A | 694 | A | -5182 | A | -51 | A |
|  | | | | | | | | |
| Well: | **B10** |  | **B11** |  | **B12** |  | **C01** |  |
| Substrates: | **L-Serine** | | **L-Threonine** | | **L-Tryptophan** | | **L-Tyrosine** | |
| DPC and ATCC strains | AUC values | Stat. different groups | AUC values | Stat. different groups | AUC values | Stat. different groups | AUC values | Stat. different groups |
| 7170 | -2695.5 | A | 2761.5 | A | -5456.5 | A | 8173.5 | A |
| 7171 | 782 | A | 3375 | A | -3978 | A | 8945 | A |
| 13518 | -2302 | A | 8743 | A | -4763.5 | A | 7371 | A |
| 13548 | -1656 | A | 14902 | A | -328.5 | A | 3783.5 | A |
| 51835 | -1718.5 | A | -635 | A | -7816.5 | A | 4028 | A |
| 6291 | -3683.5 | A | 530.5 | A | -4648.5 | A | 5650 | A |
|  | | | | | | | | |
| Well: | **C02** |  | **C03** |  | **C04** |  | **C05** |  |
| Substrates: | **L-Valine** | | **D-Alanine** | | **D-Asparagine** | | **D-Aspartic Acid** | |
| DPC and ATCC strains | AUC values | Stat. different groups | AUC values | Stat. different groups | AUC values | Stat. different groups | AUC values | Stat. different groups |
| 7170 | -486.5 | A | -4956 | A | -3634 | A | -3236 | AB |
| 7171 | 3319.5 | A | -2336 | A | -1230.5 | A | -5689 | B |
| 13518 | -1239.5 | A | -5385.5 | A | -3964.5 | A | -330 | AB |
| 13548 | -1668 | A | -4333.5 | A | -4536.5 | A | -2826 | AB |
| 51835 | -237.5 | A | -2000 | A | -824.5 | A | 323 | AB |
| 6291 | -204.5 | A | 214 | A | 2474 | A | 8881.5 | A |
|  | | | | | | | | |
| Well: | **C06** |  | **C07** |  | **C08** |  | **C09** |  |
| Substrates: | **D-Glutamic Acid** | | **D-Lysine** | | **D-Serine** | | **D-Valine** | |
| DPC and ATCC strains | AUC values | Stat. different groups | AUC values | Stat. different groups | AUC values | Stat. different groups | AUC values | Stat. different groups |
| 7170 | -24 | A | -3235.5 | A | -15189 | AB | 1161.5 | A |
| 7171 | 966 | A | -3297 | A | -21004.5 | B | 4046 | A |
| 13518 | 720 | A | -1034.5 | A | -13425.5 | AB | 1872.5 | A |
| 13548 | -1650 | A | -2983.5 | A | -6489 | AB | -1285.5 | A |
| 51835 | 1836 | A | 790.5 | A | -6431.5 | AB | 1983 | A |
| 6291 | 9136.5 | A | 1025.5 | A | -22.5 | A | 2342 | A |
|  | | | | | | | | |
| Well: | **C10** |  | **C11** |  | **C12** |  | **D01** |  |
| Substrates: | **L-Citrulline** | | **L-Homoserine** | | **L-Ornithine** | | **N-Acetyl-D,L-Glutamic Acid** | |
| DPC and ATCC strains | AUC values | Stat. different groups | AUC values | Stat. different groups | AUC values | Stat. different groups | AUC values | Stat. different groups |
| 7170 | 173 | A | -938.5 | A | -931.5 | A | -3430 | A |
| 7171 | 3102 | A | 1851 | A | -7334.5 | A | -3332.5 | A |
| 13518 | -406 | A | 6516 | A | 1002.5 | A | -5732 | A |
| 13548 | -2131.5 | A | 11177 | A | 662.5 | A | -1804 | A |
| 51835 | 1056.5 | A | -1165 | A | 1066.5 | A | -7078.5 | A |
| 6291 | 1121.5 | A | 2847 | A | 1603 | A | 1088.5 | A |
|  | | | | | | | | |
| Well: | **D02** |  | **D03** |  | **D04** |  | **D05** |  |
| Substrates: | **N-Phthaloyl-L-Glutamic Acid** | | **L-Pyroglutamic Acid** | | **Hydroxylamine** | | **Methylamine** | |
| DPC and ATCC strains | AUC values | Stat. different groups | AUC values | Stat. different groups | AUC values | Stat. different groups | AUC values | Stat. different groups |
| 7170 | -17053.5 | A | -216 | A | -5121.5 | A | -1199.5 | A |
| 7171 | -19181.5 | A | 9782 | A | -6929.5 | A | -2599 | A |
| 13518 | -18934 | A | -1527 | A | -10190.5 | A | -2010.5 | A |
| 13548 | -7745 | A | -2917.5 | A | -4252.5 | A | -3799.5 | A |
| 51835 | -21842.5 | A | 1641.5 | A | -6041 | A | 1136.5 | A |
| 6291 | -9783.5 | A | 984.5 | A | -1423 | A | 1634 | A |
|  | | | | | | | | |
| Well: | **D06** |  | **D07** |  | **D08** |  | **D09** |  |
| Substrates: | **N-Amylamine** | | **N-Butylamine** | | **Ethylamine** | | **Ethanolamine** | |
| DPC and ATCC strains | AUC values | Stat. different groups | AUC values | Stat. different groups | AUC values | Stat. different groups | AUC values | Stat. different groups |
| 7170 | -713 | A | 78 | A | -199.5 | A | 815 | A |
| 7171 | -587.5 | A | -696.5 | A | -1154.5 | A | 4597.5 | A |
| 13518 | -1825 | A | 176.5 | A | 331 | A | -211 | A |
| 13548 | -3100.5 | A | -2953.5 | A | -2582.5 | A | -1540.5 | A |
| 51835 | 1709.5 | A | 2104 | A | 1873.5 | A | 1752.5 | A |
| 6291 | 632 | A | 1510.5 | A | 1036.5 | A | 1602 | A |
|  | | | | | | | | |
| Well: | **D10** |  | **D11** |  | **D12** |  | **E01** |  |
| Substrates: | **Ethylenediamine** | | **Putrescine** | | **Agmatine** | | **Histamine** | |
| DPC and ATCC strains | AUC values | Stat. different groups | AUC values | Stat. different groups | AUC values | Stat. different groups | AUC values | Stat. different groups |
| 7170 | 352 | A | 4170.5 | A | 1370 | A | -1214 | A |
| 7171 | 2902.5 | A | 5813 | A | 1874 | A | 1655 | A |
| 13518 | -987.5 | A | 3498 | A | 5160 | A | -3229.5 | A |
| 13548 | -121 | A | 2479 | A | 2296 | A | -3018 | A |
| 51835 | 496.5 | A | 3667 | A | 13.5 | A | 2032 | A |
| 6291 | 7000 | A | 4529.5 | A | -972.5 | A | 1740.5 | A |
|  | | | | | | | | |
| Well: | **E02** |  | **E03** |  | **E04** |  | **E05** |  |
| Substrates: | **b-Phenylethylamine** | | **Tyramine** | | **Acetamide** | | **Formamide** | |
| DPC and ATCC strains | AUC values | Stat. different groups | AUC values | Stat. different groups | AUC values | Stat. different groups | AUC values | Stat. different groups |
| 7170 | -2502 | A | -629 | A | 502.5 | A | 1019 | A |
| 7171 | -2217 | A | -1645.5 | A | -646.5 | A | -260 | A |
| 13518 | -6171 | A | -3979.5 | A | -265 | A | 835.5 | A |
| 13548 | -5622.5 | A | -4942.5 | A | -2654 | A | -2159 | A |
| 51835 | -346 | A | 857 | A | 2044.5 | A | 2513 | A |
| 6291 | -1137 | A | -262 | A | 2670.5 | A | 2699.5 | A |
|  | | | | | | | | |
| Well: | **E06** |  | **E07** |  | **E08** |  | **E09** |  |
| Substrates: | Glucuronamide | | D,L-Lactamide | | D-Glucosamine | | D-Galactosamine | |
| DPC and ATCC strains | AUC values | Stat. different groups | AUC values | Stat. different groups | AUC values | Stat. different groups | AUC values | Stat. different groups |
| 7170 | 121.5 | A | -286 | A | -4338 | A | 1435.5 | A |
| 7171 | 214.5 | A | -1022.5 | A | -4797 | A | 3760.5 | A |
| 13518 | 28.5 | A | -1285 | A | -4352 | A | 377 | A |
| 13548 | -3268.5 | A | -2871.5 | A | -4381 | A | 153 | A |
| 51835 | 2536.5 | A | 1875.5 | A | -1623 | A | 2110.5 | A |
| 6291 | 1433 | A | -210.5 | A | -3747.5 | A | 1888 | A |
|  | | | | | | | | |
| **Well:** | **E10** |  | **E11** |  | **E12** |  | **F01** |  |
| Substrates: | **D-Mannosamine** | | **N-Acetyl-D-Glucosamine** | | **N-Acetyl-D-Galactosamine** | | **N-Acetyl-D-Mannosamine** | |
| DPC and ATCC strains | AUC values | Stat. different groups | AUC values | Stat. different groups | AUC values | Stat. different groups | AUC values | Stat. different groups |
| 7170 | -1252 | A | 1402 | A | 1925.5 | A | 1099 | A |
| 7171 | -2365 | A | 1448 | A | 600.5 | A | -463 | A |
| 13518 | -121.5 | A | 1623.5 | A | 1996 | A | 394.5 | A |
| 13548 | 1412 | A | -434 | A | 1420.5 | A | -3390.5 | A |
| 51835 | 153.5 | A | 2464 | A | 1632.5 | A | 1562.5 | A |
| 6291 | 1372.5 | A | 1629.5 | A | 3375.5 | A | 3061 | A |
|  | | | | | | | | |
| **Well:** | **F02** |  | **F03** |  | **F04** |  | **F05** |  |
| Substrates: | **Adenine** | | **Adenosine** | | **Cytidine** | | **Cytosine** | |
| DPC and ATCC strains | AUC values | Stat. different groups | AUC values | Stat. different groups | AUC values | Stat. different groups | AUC values | Stat. different groups |
| 7170 | -10289 | A | -10343 | A | 4776 | A | -2441 | A |
| 7171 | -7081.5 | A | -9617.5 | A | 5120 | A | -3246 | A |
| 13518 | -12698.5 | A | -15394 | A | -1363.5 | A | -3243.5 | A |
| 13548 | -7816.5 | A | -8280.5 | A | -6054.5 | A | -7886 | A |
| 51835 | -11378 | A | -17152 | A | 364 | A | -76.5 | A |
| 6291 | -8142 | A | -7499 | A | 5771 | A | -2584.5 | A |
|  | | | | | | | | |
| Well: | **F06** |  | **F07** |  | **F08** |  | **F09** |  |
| Substrates: | **Guanine** | | **Guanosine** | | **Thymine** | | **Thymidine** | |
| DPC and ATCC strains | AUC values | Stat. different groups | AUC values | Stat. different groups | AUC values | Stat. different groups | AUC values | Stat. different groups |
| 7170 | -15573 | A | -3916.5 | A | -1534 | A | -3395.5 | A |
| 7171 | -14875 | A | -5190.5 | A | -1912 | A | -9019 | A |
| 13518 | -14139.5 | A | -9348.5 | A | -2042.5 | A | -6609 | A |
| 13548 | -6354.5 | A | -6975.5 | A | -6371.5 | A | -4386 | A |
| 51835 | -21901.5 | A | -2825.5 | A | -1288 | A | -2453 | A |
| 6291 | -7138 | A | 1251 | A | -1051.5 | A | 3498.5 | A |
|  | | | | | | | | |
| Well: | **F10** |  | **F11** |  | **F12** |  | **G01** |  |
| Substrates: | **Uracil** | | **Uridine** | | **Inosine** | | **Xanthine** | |
| DPC and ATCC strains | AUC values | Stat. different groups | AUC values | Stat. different groups | AUC values | Stat. different groups | AUC values | Stat. different groups |
| 7170 | 2188 | A | 2134 | A | 771 | A | 4688.5 | A |
| 7171 | 810 | A | 2453 | A | 120 | A | 4216.5 | A |
| 13518 | 2021.5 | A | -2012 | A | -3715 | A | 2164 | A |
| 13548 | 166.5 | A | -433.5 | A | -368 | A | 5927.5 | A |
| 51835 | 1225 | A | 382.5 | A | -1271 | A | 2797.5 | A |
| 6291 | 3052 | A | 3039.5 | A | 3321 | A | 5945 | A |
|  | | | | | | | | |
| Well: | **G02** |  | **G03** |  | **G04** |  | **G05** |  |
| Substrates: | **Xanthosine** | | **Uric Acid** | | **Alloxan** | | **Allantoin** | |
| DPC and ATCC strains | AUC values | Stat. different groups | AUC values | Stat. different groups | AUC values | Stat. different groups | AUC values | Stat. different groups |
| 7170 | -3584.5 | A | -6491 | A | -2463.5 | A | 91.5 | A |
| 7171 | -4902.5 | A | -8565.5 | A | -4356 | A | -1452 | A |
| 13518 | -6671 | A | -7033.5 | A | -4813 | A | -101 | A |
| 13548 | -1456.5 | A | -5423.5 | A | 5778 | A | 367.5 | A |
| 51835 | -2725.5 | A | -4348.5 | A | -6159.5 | A | 161 | A |
| 6291 | -3026.5 | A | 413.5 | A | 7883.5 | A | 2332.5 | A |
|  | | | | | | | | |
| Well: | **G06** |  | **G07** |  | **G08** |  | **G09** |  |
| Substrates: | **Parabanic Acid** | | **D,L-a-Amino-N-Butyric Acid** | | **g-Amino-N-Butyric Acid** | | **e-Amino-N-Caproic Acid** | |
| DPC and ATCC strains | AUC values | Stat. different groups | AUC values | Stat. different groups | AUC values | Stat. different groups | AUC values | Stat. different groups |
| 7170 | -3510.5 | A | -4000.5 | A | -612 | A | 1750 | A |
| 7171 | -7387 | A | 575.5 | A | 1250 | A | 872.5 | A |
| 13518 | -4159 | A | -4096.5 | A | -1280 | A | 3259.5 | A |
| 13548 | -3083 | A | -3038 | A | -2802 | A | 631.5 | A |
| 51835 | -4000.5 | A | -2240 | A | 450.5 | A | 2074 | A |
| 6291 | -686.5 | A | 1105.5 | A | 833 | A | 2336.5 | A |
|  | | | | | | | | |
| Well: | **G10** |  | **G11** |  | **G12** |  | **H01** |  |
| Substrates: | **D,L-a-Amino-Caprylic Acid** | | **d-Amino-N-Valeric Acid** | | **a-Amino-N-Valeric Acid** | | **Ala-Asp** | |
| DPC and ATCC strains | AUC values | Stat. different groups | AUC values | Stat. different groups | AUC values | Stat. different groups | AUC values | Stat. different groups |
| 7170 | 17846.5 | A | 1677.5 | A | -730 | A | -2245 | A |
| 7171 | 16841 | A | 1561 | A | -2987 | A | -3574 | A |
| 13518 | 19034.5 | A | 2229.5 | A | 228 | A | -2292.5 | A |
| 13548 | 29853 | A | 1223 | A | 2704 | A | 5666 | A |
| 51835 | 10269 | A | 2048.5 | A | -1403 | A | -2519 | A |
| 6291 | 27382 | A | 3093 | A | 3707.5 | A | 10663.5 | A |
|  | | | | | | | | |
| Well: | H02 |  | H03 |  | H04 |  | H05 |  |
| Substrates: | **Ala-Gln** | | **Ala-Glu** | | **Ala-Gly** | | **Ala-His** | |
| DPC and ATCC strains | AUC values | Stat. different groups | AUC values | Stat. different groups | AUC values | Stat. different groups | AUC values | Stat. different groups |
| 7170 | -2789 | A | -704.5 | A | 9923.5 | A | 744 | A |
| 7171 | -38.5 | A | -3775.5 | A | 9674.5 | A | 2810.5 | A |
| 13518 | -4879 | A | 1680 | A | 5198 | A | 1025.5 | A |
| 13548 | -1177.5 | A | 2412 | A | 9817.5 | A | 3612.5 | A |
| 51835 | -1357.5 | A | 1461.5 | A | -1679.5 | A | 1640.5 | A |
| 6291 | 989.5 | A | 9172 | A | 11185 | A | 1957.5 | A |
|  | | | | | | | | |
| Well: | **H06** |  | **H07** |  | **H08** |  | **H09** |  |
| Substrates: | Ala-Leu | | Ala-Thr | | Gly-Asn | | Gly-Gln | |
| DPC and ATCC strains | AUC values | Stat. different groups | AUC values | Stat. different groups | AUC values | Stat. different groups | AUC values | Stat. different groups |
| 7170 | -4795 | A | 8869.5 | A | -12281.5 | A | -376.5 | A |
| 7171 | -1364 | A | 7135 | A | -12220 | A | 3519 | A |
| 13518 | -8629 | A | 9565 | A | -12500.5 | A | -4757 | A |
| 13548 | -2624 | A | 17290.5 | A | -5050 | A | 1639.5 | A |
| 51835 | -4725 | A | -1798.5 | A | -6187 | A | -4428 | A |
| 6291 | -1770.5 | A | 13119 | A | -4098 | A | 11358 | A |
|  | | | | | | |  |  |
| Well: | **H10** |  | **H11** |  | **H12** |  |  |  |
| Substrates: | **Gly-Glu** | | **Gly-Met** | | **Met-Ala** | |  |  |
| DPC and ATCC strains | AUC values | Stat. different groups | AUC values | Stat. different groups | AUC values | Stat. different groups |  |  |
| 7170 | 7650 | A | 5704.5 | A | 4480.5 | A |  |  |
| 7171 | 7099.5 | A | 8550.5 | A | 5745.5 | A |  |  |
| 13518 | 11028 | A | 8485.5 | A | 6599 | A |  |  |
| 13548 | 14952 | A | 14067.5 | A | 8770.5 | A |  |  |
| 51835 | 1992 | A | -527 | A | 608 | A |  |  |
| 6291 | 15401 | A | 1556.5 | A | 934.5 | A |  |  |
