# Supplementary material for: A Systems-Wide Analysis of Proteolytic and Lipolytic Pathways Uncovers The Flavor-Forming Potential of The Gram-Positive Bacterium Macrococcus caseolyticus subsp. caseolyticus
Source: Front Microbiol. 2020 Jul 7;11:1533. doi: 10.3389/fmicb.2020.01533 (PMC7358451; doi:10.3389/fmicb.2020.01533)
Supplement: TABLE S5 — Volatiles detected with HS-SPME GCMS absent in control and associated with test strains, reported with relative identification CAS number and the linear retention index (LRI). All results presented are obtained in this study. [file Table_5.DOCX]

**Table S5**: Volatiles detected with HS-SPME GCMS absent in control and associated with test strains, reported with relative identification CAS number and the linear retention index (LRI). All results presented are obtained in this study.

| **Compounds** | **CAS** | **LRI** | **DPC6291** | **DPC7170** | **DPC7171** | **ATCC13548** | **ATCC13518** | **ATCC51835** |
| --- | --- | --- | --- | --- | --- | --- | --- | --- |
| **Trial 1** | | | | | | | | |
| Methyl butanoate | 623427 | 748 | +* | + | + | + | + | + |
| Propyl butanoate | 644495 | 922 | +* | + | + | + | + | + |
| Methyl hexanoate | 106707 | 949 | ND | ND | ND | ND | +* | +* |
| Butyl butanoate | 109217 | 1019 | ND | +* | +* | +* | ND | ND |
| Isoamyl isobutanoate | 2050013 | 1080 | ND | +* | +* | +* | ND | ND |
| 2-Methylbutyl butanoate | 51115641 | 1086 | ND | +* | +* | +* | ND | ND |
| 4-Pentenyl butyrate | 30563316 | 1093 | ND | +* | +* | +* | ND | ND |
| Amyl isobutyrate | 2445729 | 1117 | ND | +* | +* | +* | ND | ND |
| Butyl hexanoate | 626824 | 1215 | ND | +* | +* | +* | ND | ND |
| Hexyl butanoate | 2639636 | 1216 | ND | +* | ND | ND | ND | ND |
| Isopentyl hexanoate | 2198610 | 1276 | ND | +* | +* | ND | ND | ND |
| Ethyl decanoate | 110383 | 1420 | ND | ND | ND | ND | ND | +* |
| 2,3-Heptanedione | 96048 | 876 | ND | ND | ND | ND | +* | ND |
| 2-Undecanone | 112129 | 1331 | ND | ND | + | + | + | +* |
| Benzeneacetaldehyde | 122781 | 1111 | ND | ND | + | + | +* | ND |
| 3-Methyl butanal | 590863 | 692 | + | + | + | + | +* | + |
| Nonanal | 124196 | 1145 | +* | + | ND | ND | ND | ND |
| Isopropyl Alcohol | 67630 | 548 | ND | +* | ND | ND | ND | ND |
| Methanethiol | 74931 | 463 | +* | ND | ND | ND | ND | ND |
| Acetic acid | 64197 | 704 | +* | + | + | + | + | + |
| Butanoic acid | 107926 | 871 | +* | + | + | +* | + | + |
| p-Cresol | 106445 | 1182 | +* | ND | ND | ND | ND | ND |
| Phenylethyl Alcohol | 60128 | 1194 | ND | ND | ND | ND | +* | ND |

Legend: +^*^, significantly higher from other strains (P< 0.05); +, volatile detected but not significant; ND, Not detected
