# Supplementary material for: A Systems-Wide Analysis of Proteolytic and Lipolytic Pathways Uncovers The Flavor-Forming Potential of The Gram-Positive Bacterium Macrococcus caseolyticus subsp. caseolyticus
Source: Front Microbiol. 2020 Jul 7;11:1533. doi: 10.3389/fmicb.2020.01533 (PMC7358451; doi:10.3389/fmicb.2020.01533)
Supplement: TABLE S6 — Cell enumeration presented in log CFU ml–1, in Lactose free milk before (t = 0 h) and after incubation at 37°C (t = 24 h). pH values; in LFM after incubation at 37°C (t = 24 h). [file Table_6.DOCX]

Table S6: Cell enumeration presented in log CFU ml^-1^, in Lactose free milk before (t = 0 h) and after incubation at 37°C (t = 24 h). pH values; in LFM after incubation at 37°C (t = 24 h).

| **Strain** |  | | |  |
| --- | --- | --- | --- | --- |
|  | **t=0h** | **t=24h** | **pH=24** |  |
| DPC 6291 | 3.80±0.27 | 6.43±0.416 | 5.27±0.026 |  |
| ATCC 51835 | 3.58±0.02 | 6.5±0.3 | 6.376±0.14 |  |
| ATCC 13548 | 3.66±0.12 | 6.28±0.131 | 5.7±0.09 |  |
| ATCC 13518 | 3.87±0.08 | 6.5±0.264 | 6.43±0.085 |  |
| DPC 7170 | 3.22±0.65 | 6.12±0.155 | 5.5±0.1 |  |
| DPC 7171 | 3.68±0.155 | 7.2±0.1 | 5.72±0.064 |  |
| Control | ND | ND | 6.8±0.05 |  |

Results are shown as mean values ± SD of triplicate experiments. ND=not detected.
