# Supplementary material for: A Systems-Wide Analysis of Proteolytic and Lipolytic Pathways Uncovers The Flavor-Forming Potential of The Gram-Positive Bacterium Macrococcus caseolyticus subsp. caseolyticus
Source: Front Microbiol. 2020 Jul 7;11:1533. doi: 10.3389/fmicb.2020.01533 (PMC7358451; doi:10.3389/fmicb.2020.01533)
Supplement: TABLE S7 — Flavor contributing Volatiles significantly associated with M. caseolyticus subsp. caseolyticus strains (P < 0.05). [file Table_7.DOCX]

| **Compound** | **Flavour description** | **DPC6291** | **DPC7170** | **DPC7171** | **ATCC 51835** | **ATCC13548** | **ATCC 13518** | **RI** |
| --- | --- | --- | --- | --- | --- | --- | --- | --- |
| **Acid** |  |  |  |  |  |  |  |  |
| Acetic acid | Vinegar, peppers, green, fruity floral, sour | + | +* | + | ND | +* | + | 689 |
| Butanoic acid | Sweaty, butter, cheese, strong, acid, rancid, dirty sock | +* | + | + | + | +* | + | 866 |
| **Aldehyde** |  |  |  |  |  |  |  |  |
| 3-Methyl-butanal | Malty, dark chocolate, almond, cocoa, coffee | + | + | + | + | + | +* | 693 |
| Nonanal | Green, citrus, fatty, floral | +* | + | + | ND | ND | ND | 1148 |
| **Ketone** |  |  |  |  |  |  |  |  |
| 2-Undecanone | Floral,fruity, green, musty, tallow | ND | ND | + | +* | + | +* | 1330 |
| **Ester** |  |  |  |  |  |  |  |  |
| Ethyl decanoate | "Queso de flor" ("FLOR" CHEESE) | ND | ND | ND | +* | ND | ND | 1419 |
| Methyl butanoate | Sweet, fruity | + | + | +* | ND | + | + | 748 |
| Butyl butanoate | Pineapple, banana, sweet | ND | +* | +* | ND | +* | ND | 1019 |
| Butyl hexanoate | Fruity, pineapple, waxy, green, juicy, apple | ND | +* | +* | ND | +* | ND | 1215 |
| **Phenolic compounds** |  |  |  |  |  |  |  |  |
| p-Cresol | Cowy-barny | +* | ND | ND | ND | ND | + | 1182 |
| **Sulfur compound** |  |  |  |  |  |  |  |  |
| Methanethiol | Rotting cabbage, cheese, vegetative, sulphur | +* | ND | ND | ND | ND | ND | 460 |

Table S7: Flavour contributing Volatiles significantly associated with *M. caseolyticus* subsps *caseolyticus* strains (P< 0.05).

Legend: +*, significantly higher from other strains (P< 0.05); +, volatile detected but not significant; ND, Not detected
